# Atomically thin vanadium oxide nanosheets *via* top-down exfoliation for ultrafast-charging zinc-ion batteries

**DOI:** 10.1039/d6sc05963e

**Published:** 2026-09-07

**Authors:** Weikang Jiang, Xiling Niu, Kaiyue Zhu, Weili Xie, Zhengsen Wang, Weishen Yang

**Affiliations:** a State Key Laboratory of Catalysis, Dalian Institute of Chemical Physics, Chinese Academy of Sciences Dalian 116023 China yangws@dicp.ac.cn zky218@dicp.ac.cn; b University of Chinese Academy of Sciences Beijing 100049 China; c Henan Key Laboratory of Quantum Materials and Quantum Energy, School of Quantum Information Future Technology, Henan University Kaifeng 475001 China

## Abstract

Aqueous zinc-ion batteries (ZIBs) are a promising energy storage solution due to their high safety, environmental friendliness and low cost. Although the high ionic conductivity of aqueous electrolytes and fast kinetics of zinc plating/stripping hold promise for fast discharge/charge processes, the rate capability of AZIBs remains limited due to poor ion and electron transport in cathode materials. This study addresses this challenge by developing atomically thin vanadium oxide nanosheets (∼2 nm thickness) through a top-down exfoliation strategy using bulk ZnV_6_O_16_·*n*H_2_O. Driven by small-molecule-assisted interlayer interactions, a mild wet ball-milling and ultrasonication process effectively disrupts interlayer hydrogen bonding and enables efficient layer separation. Benefiting from the reduced thickness (layers) and weakened interlayer interactions, the exfoliated ZnV_6_O_16_·*n*H_2_O nanosheets deliver a solid-state ion diffusion coefficient nearly eight times higher than the bulk, together with a decreased diffusion barrier (84.6 meV *versus* 187.5 meV), promoting rapid charge storage. Finally, a high capacity of 346 mAh g^−1^ (4.5 times that of the bulk) at 50 A g^−1^ and excellent cycling stability (77% capacity retention after 30 000 cycles at 100 A g^−1^) are achieved in coin-type cells based on ultrathin ZVO nanosheets. Moreover, a 45 cm^2^ pouch cell maintains 98% capacity retention over 550 cycles, highlighting the strong potential for scalable preparation and practical application. These findings demonstrate a promising strategy for designing high-performance, fast-charging batteries, advancing grid-scale energy storage solutions.

## Abstract 2

Atomically thin vanadium oxide nanosheets were synthesized *via* a mild liquid-phase top-down exfoliation method and then integrated with few-layer graphene, yielding exceptional rate performance and cycling stability in aqueous zinc-ion batteries.

## Graphical abstract

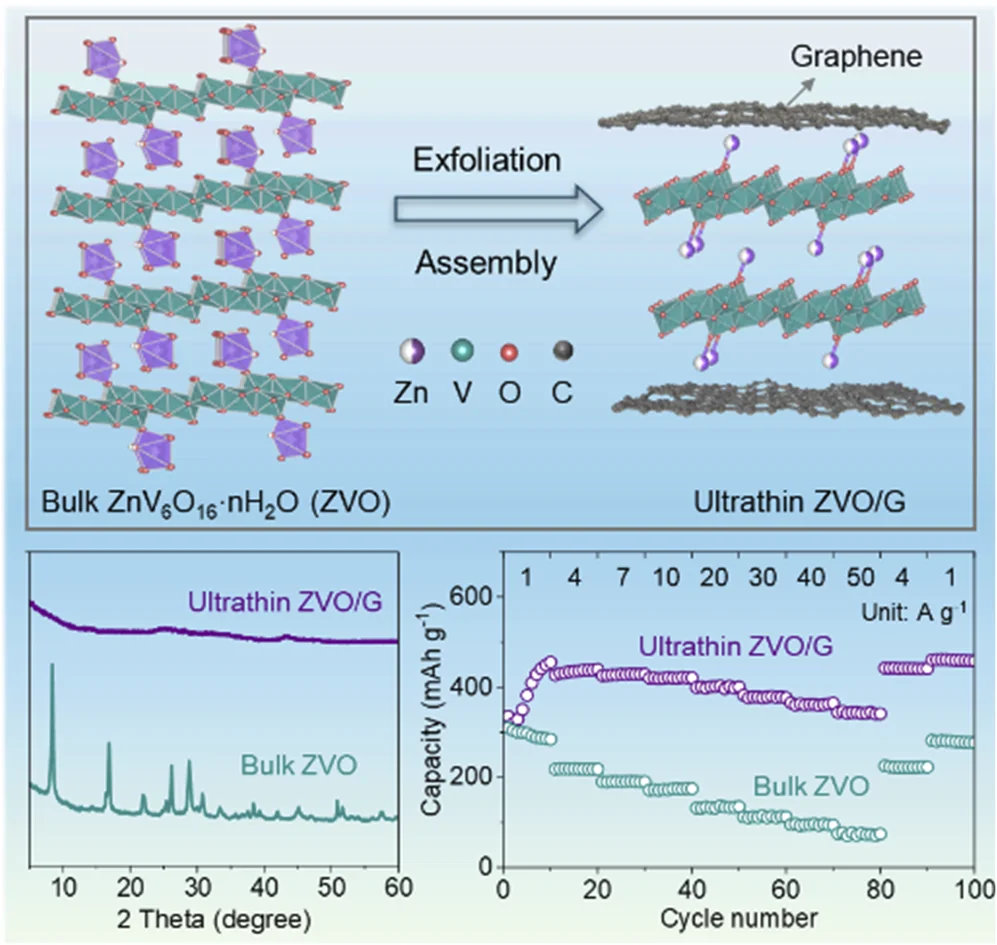

## Introduction

As the global energy landscape shifts from fossil fuels to renewables, efficient energy storage systems are essential to fully harness intermittent sources like solar and wind.^1,2^ Rechargeable batteries play a key role in this transition, enabling reliable energy storage and delivery with rapid response capabilities. While lithium-ion batteries (LIBs) have revolutionized the commercial battery market, their reliance on flammable and relatively low-conductivity organic electrolytes, together with the scarcity of critical raw materials, has led to safety concerns, high costs and limited fast-charging capabilities. These limitations hinder their widespread application in grid-scale energy storage. In contrast, rechargeable aqueous zinc-ion batteries (AZIBs) offer intrinsic safety, low cost, and environmental friendliness,^3–7^ making them attractive for grid-scale energy storage. Benefiting from fast ionic transport in aqueous electrolytes, AZIBs exhibit faster ion transport and potentially superior charging capabilities compared with LIBs. However, AZIBs still fall short of delivering true ultrafast charging, limiting their broader deployment.^8–10^ The main bottleneck is the sluggish reaction kinetics in cathode materials, resulting in a sharp capacity drop at high charge/discharge rates.^11,12^ To address this, developing cathodes with ultrafast charge-storage capabilities is essential for enabling high power outputs and shortening charging times.

Common AZIB cathodes include manganese- and vanadium-based oxides, Prussian blue analogues, and organic compounds.^13–16^ Among them, vanadium-based oxides stand out for their high theoretical capacities due to their multiple oxidation states from +3 to +5, and their excellent cycling stability owing to their layered structures. However, their practical rate capability remains unsatisfactory because the limited ion diffusion kinetics and intrinsically poor electronic conductivity within bulk materials restrict active-site accessibility, resulting in incomplete utilization of the available charge-storage sites.^11,17,18^ To overcome these issues, previous efforts have mainly focused on widening interlayer spacing and adding conductive components to facilitate ion diffusion and electron transport. For example, various cations (*e.g.*, Zn^2+^, Ca^2+^, and NH_4_^+^) and molecules (*e.g.*, H_2_O and polyaniline) have been intercalated into vanadium-based oxide layers to enlarge the interlayer distance and accelerate ion transport within the host structure.^19–22^ Similarly, conductive polymers like polyaniline and poly(3,4-ethylenedioxythiophene) (PEDOT), as well as multilayer graphene, have been mixed with vanadium-based oxides to improve electronic conductivity.^23–28^ However, even with these modifications, vanadium-based cathodes typically remain in bulk form, which limits the effective utilization of interior active sites. Strong interlayer interactions and poor intrinsic electronic conductivity render a large fraction of active sites electrochemically inaccessible during fast charge/discharge processes. Reducing the number of layers and decreasing material thickness represents a promising strategy to maximize active-site exposure. Theoretically, few-layer or atomically thin structures can significantly shorten ion diffusion and electron transport pathways, thereby accelerating charge-storage kinetics. However, conventional synthesis approaches, such as hydrothermal and electrochemical methods, usually produce multilayered crystalline materials, making the controllable bottom-up construction of atomically thin single-layer or few-layer vanadium-based structures highly challenging.^29,30^

Here, we present a top-down exfoliation strategy to address this challenge. As a proof-of-concept, layered vanadium oxide (ZnV_6_O_16_·*n*H_2_O, ZVO) was successfully exfoliated for the first time into ultrathin nanosheets with atomic-scale thickness (∼2 nm) through a novel, mild liquid-phase exfoliation method. This strategy effectively converts the electrochemically inaccessible interior layers of bulk ZVO into highly exposed active surfaces, substantially enhancing interlayer ion-transport kinetics. Furthermore, to enhance electronic conductivity and prevent ZVO nanosheets from restacking during electrode preparation, few-layer graphene was integrated with ultrathin ZVO nanosheets *via* an electrostatic self-assembly process, yielding a robust ZVO/graphene heterostructure. This design allows the few-layer ZVO nanosheet cathode to achieve a solid-state ion diffusion coefficient nearly eight times higher than that of bulk ZVO, together with a markedly reduced ion diffusion barrier (84.6 meV *versus* 187.5 meV). Benefiting from the highly exposed vanadium active sites that enable robust, fast multielectron redox reactions and efficient charge transfer, the ultrathin ZVO nanosheets outperform the bulk cathode material, delivering superior high-rate capability and remarkable long-term cycling stability in both coin- and pouch-type AZIBs.

## Results and discussion

### Formation of atomically thin vanadium oxide nanosheets

**Fig. 1:**
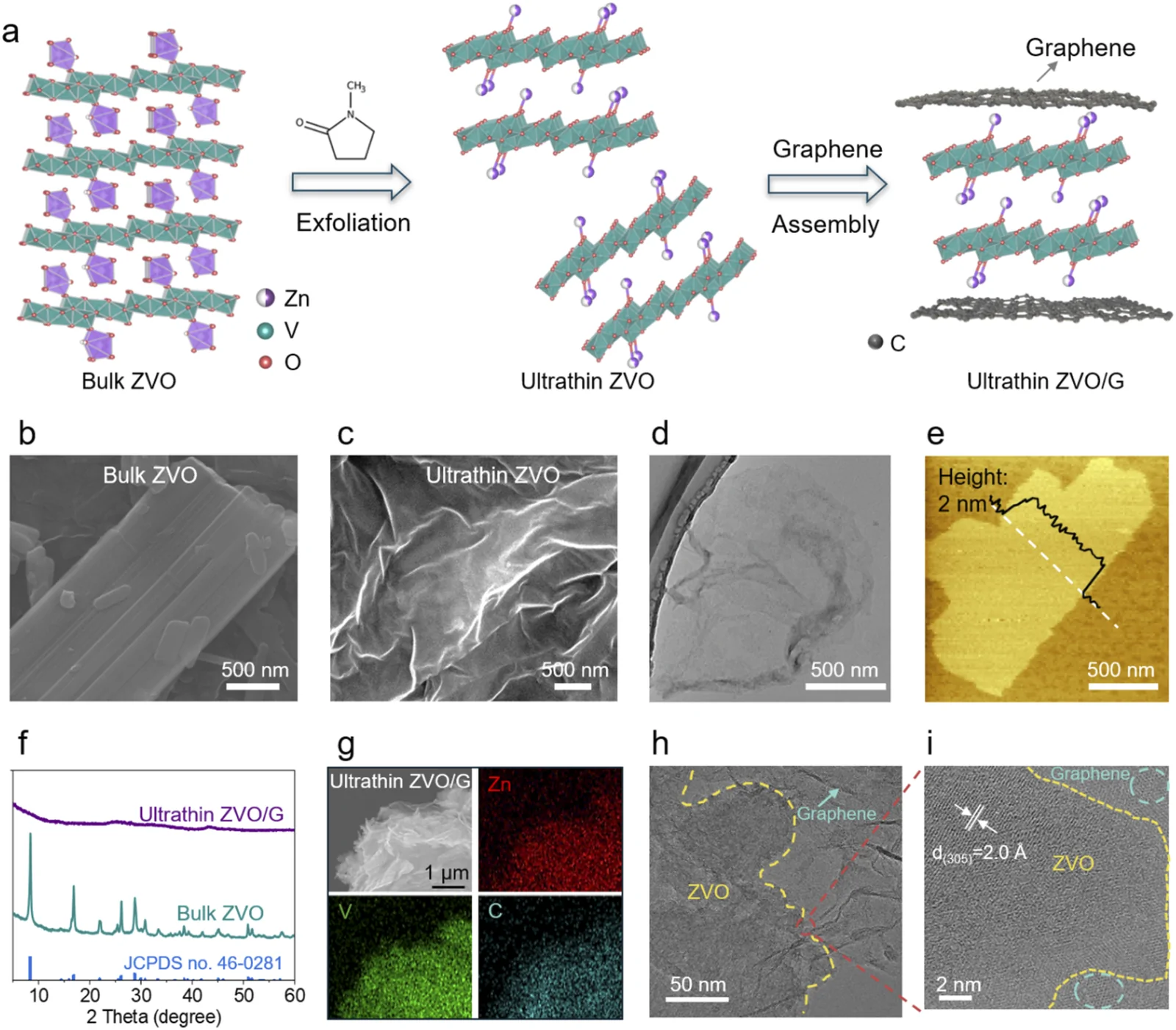
The formation of the ultrathin ZVO/G heterostructure. (a) A schematic illustration of NMP-assisted exfoliation and assembly from bulk ZVO to ultrathin ZVO/G. (b) An SEM image of bulk ZVO. (c–e) SEM, TEM, and AFM images of ultrathin ZVO nanosheets, respectively. (f) XRD patterns of bulk ZVO and ultrathin ZVO/G. (g) A scanning transmission electron microscopy (STEM) image with corresponding element mapping. (h) A TEM image and (i) high-resolution TEM image of ultrathin ZVO/G. The lattice spacing of 2.0 Å corresponds to the (305) plane of ZVO.

Hewettite ZnV_6_O_16_·*n*H_2_O (ZVO) was selected as the layered vanadium-based precursor due to its large interlayer spacing and water-accommodating structure. In this hewettite structure (Fig. 1a, left), ZVO consists of two-dimensional (2D) vanadium oxide layers stacked along the *c*-axis and interconnected through hydrogen bonding interactions.^31^ Each vanadium ion in the layers is coordinated by six oxygen atoms, forming a distorted octahedral [VO_6_] geometry. The as-synthesized bulk ZVO exhibits a stacked nanobelt morphology (Fig. 1b and S1). Although hydrated ions and water molecules can be incorporated into the interlayer galleries, thus expanding the gallery spacing of bulk ZVO, the strong hydrogen-bonding interactions between adjacent layers maintain a tightly stacked bulk structure. To overcome these interlayer interactions and achieve efficient layer separation, a mechanical-force-assisted exfoliation method was developed. Specifically, ZVO was first subjected to wet ball-milling in *N*-methyl-2-pyrrolidone (NMP), during which NMP molecules penetrated the interlayer galleries and replaced the intercalated water molecules, thereby weakening the hydrogen-bonding interactions between neighboring layers. The resulting dispersion was subsequently treated by ultrasonication to further promote layer delamination and generate ultrathin ZVO nanosheets (Fig. 1a, center). The exfoliated ZVO dispersion exhibits a pronounced Tyndall effect (Fig. S2), confirming the formation of a stable and homogeneous colloidal suspension of ultrathin nanosheets. Notably, the bulk ZVO dispersion exhibits noticeable precipitation at the bottom (Fig. S3), which can be attributed to the relatively large particle size and severe agglomeration of bulk ZVO. After 5 h of immersion, the amount of precipitate further increases, accompanied by a weakened Tyndall effect, indicating the poor dispersion stability of the unexfoliated bulk ZVO.

The scanning electron microscopy (SEM) images show that the original stacked nanobelts (Fig. 1b) were fully transformed into ultrathin nanosheets after the top-down exfoliation process (Fig. 1c and S4). Transmission electron microscopy (TEM) images (Fig. 1d, S5 and S6) further reveal the formation of flexible and highly wrinkled nanosheets, characteristic of ultrathin two-dimensional (2D) structures. Atomic force microscopy (AFM) measurements in tapping mode (Fig. 1e) show that an individual ZVO nanosheet possesses lateral dimensions of up to ∼2 µm and a thickness of ∼2 nm, corresponding to approximately two stacked [V_6_O_16_]^2−^ layers considering a (001) interlayer spacing of approximately 1.04 nm. Brunauer–Emmett–Teller (BET) analysis further confirms the transformation, with the specific surface area increasing markedly from 19 m^2^ g^−1^ for bulk ZVO to 77 m^2^ g^−1^ for ultrathin ZVO nanosheets after top-down exfoliation (Fig. S7), implying the substantially enhanced exposure of electrochemically active sites. Notably, Fig. S2 and S7 demonstrate that the ultrathin ZVO suspension in NMP remains stable with no observable precipitate even after one month of standing, clearly indicating effective exfoliation. Moreover, the exfoliation process is not limited to NMP solvent. Other polar solvents, such as dimethylformamide (DMF) and dimethylacetamide (DMA), can also effectively facilitate ZVO exfoliation, whereas solvents such as acetonitrile, acetone and alcohols (methanol, ethanol, and isopropanol) show limited or negligible exfoliation capabilities (Fig. S8–S10).

### Assembly of atomically thin vanadium oxide nanosheets with few-layer graphene

To prevent the restacking (Fig. S11) of ultrathin ZVO nanosheets during drying and to simultaneously enhance their intrinsically poor electronic conductivity, these exfoliated nanosheets were integrated with conductive few-layer graphene (Fig. S12). The ZVO nanosheets carry a negative surface charge, while the graphene nanosheets possess a positively charged surface, as confirmed by zeta potential measurements (Fig. S13). These opposite surface charges enable strong electrostatic attraction between the two components. Through a solvothermal-assisted electrostatic self-assembly process (Fig. 1a, right), a well-integrated heterostructure, referred to as ultrathin ZVO/G, was successfully constructed. X-ray diffraction (XRD) analysis (Fig. 1f) reveals the disappearance of the characteristic diffraction peaks associated with bulk ZVO in the ultrathin ZVO/G composite, confirming the effective exfoliation of the layer structure and the suppression of nanosheet restacking.

As shown in Fig. S14, the (001) peak intensity gradually decreases upon increasing the ball-milling time of ZVO in NMP from 0 h to 6 h, indicating a reduction in the number of layers. This is primarily caused by the combined effects of a reduction in the coherent scattering volume of the crystal and weakened interlayer interference, arising from both reduced out-of-plane ordering due to interlayer gliding and a size/thickness effect from the ultrathin nanosheets. Concurrently, the (001) peak shifts to a higher diffraction angle, suggesting the intercalation of NMP molecules into the ZVO interlayers, where they replace the original interlayer H_2_O. Unlike water, which forms strong hydrogen bonds with the host layers and accommodates a multilayer water structure, NMP interacts only weakly with the layers and lacks hydrogen-bonding capabilities. Consequently, probably only a single layer of NMP molecules remains intercalated between the layers after drying, leading to a decrease in the interlayer spacing.

Furthermore, elemental mapping (Fig. 1g and S15) confirms the uniform dispersion of Zn, V, O, and C throughout the ultrathin ZVO/G composite, indicating the homogeneous integration of ultrathin ZVO nanosheets with graphene.

TEM and SEM images (Fig. 1h and S16) reveal a characteristic folded sheet-like morphology, further confirming the formation of ultrathin layered structures. High-resolution TEM imaging (Fig. 1i) clearly demonstrates the intimate contact and strong interfacial coupling between the ZVO nanosheets and graphene. In contrast, a control sample created by simply ball-milling bulk ZVO with graphene (ZVO/G-BM) exhibits no significant morphological evolution or ultrathin nanosheet formation (Fig. S17), highlighting that the prior exfoliation step is essential for achieving effective layer thinning and subsequent interfacial assembly.

Spectroscopic analyses further verify strong interfacial interactions between ZVO nanosheets and graphene in the heterostructure. X-ray photoelectron spectroscopy (XPS, Fig. S18) shows the emergence of new C–O bonds in ultrathin ZVO/G, which are absent in both bulk ZVO and ZVO/G-BM, indicating the formation of interfacial chemical bonding between exfoliated ZVO nanosheets and graphene in the ultrathin ZVO/G heterostructure. In addition, the broadened O 1s and V 2p signals further indicate the presence of altered local electronic environments associated with interfacial bonding. Raman spectroscopy (Fig. S19) further supports the enhanced interaction, with the V–O stretching mode in ZVO/G exhibiting a noticeable redshift compared to bulk ZVO, providing additional evidence for strengthened interaction between ZVO nanosheets and graphene. Compositional analysis using inductively coupled plasma optical emission spectrometry (ICP-OES) and thermal gravimetric analysis (TGA) (Table S1 and Fig. S20) shows that the ultrathin ZVO/G composite maintains a Zn : V atomic ratio of 0.9 : 6, consistent with the original hewettite stoichiometry, and possesses ∼2.2H_2_O per formula unit, with graphene making up approximately 16 wt% of the total mass.

This work successfully exfoliates bulk ZVO into atomically thin (∼2 nm) nanosheets and subsequently assembles them with few-layer graphene to form a highly conductive and structurally stable ultrathin ZVO/G heterostructure. The top-down exfoliation process substantially increases the surface area and exposes more redox-active vanadium sites, while the integration with graphene simultaneously enhances electronic conductivity and effectively suppresses nanosheet restacking. These synergistic structural and compositional advantages establish a favorable framework for accelerated ion/electron transport and enhanced electrochemical reactions, highlighting ultrathin ZVO/G as a promising cathode material for high-performance zinc-ion batteries.

### The electrochemical performance of ultrathin ZVO/G in AZIBs

**Fig. 2:**
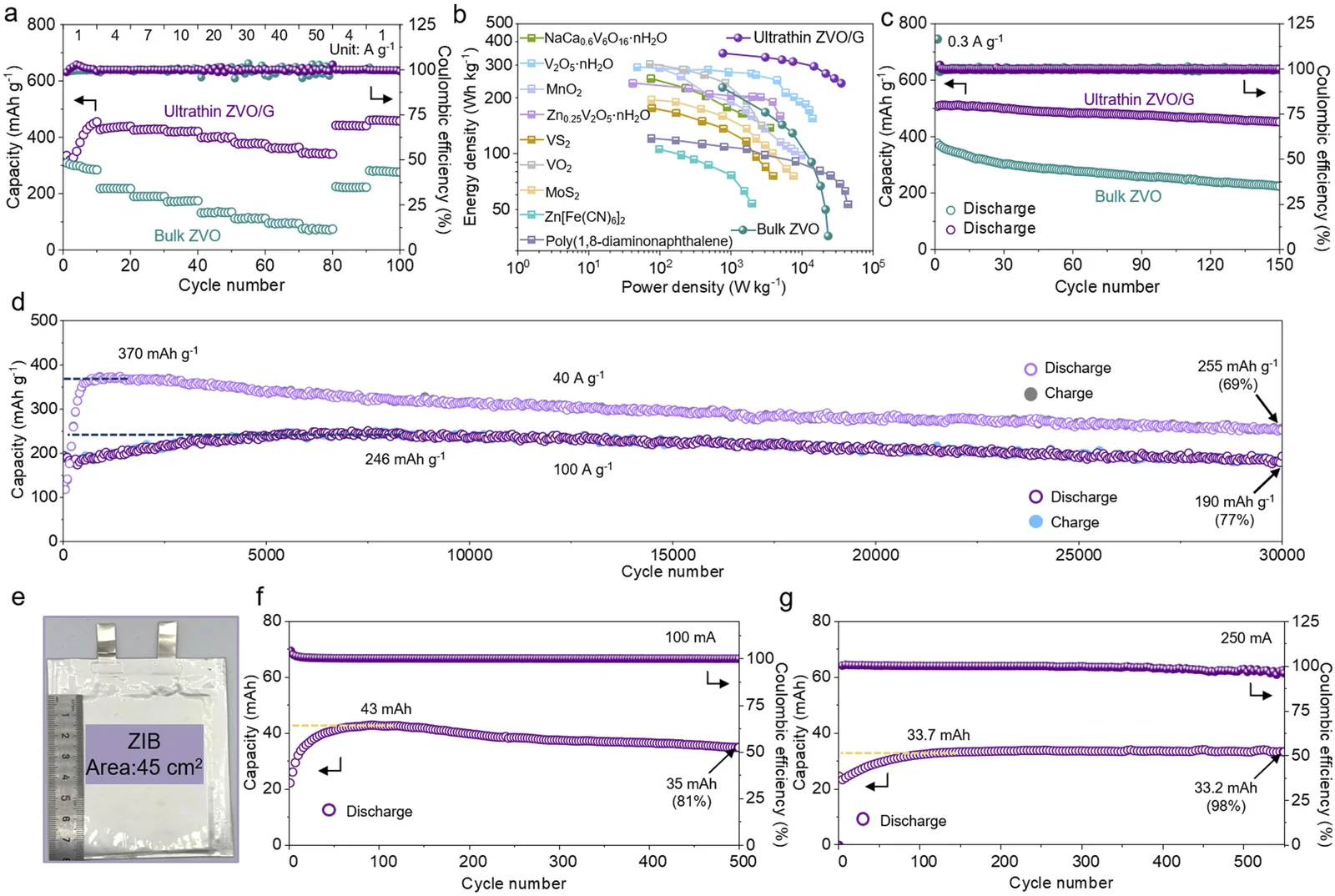
The superior electrochemical performance of ultrathin ZVO/G in AZIBs. (a) The rate performance of bulk ZVO *vs.* ultrathin ZVO/G. (b) A Ragone plot comparing ultrathin ZVO/G with other representative AZIB cathodes (energy and power densities calculated based on the mass of cathode active material). (c) The cycling performance at 0.3 A g^−1^ for ultrathin ZVO/G and bulk ZVO. (d) The cycling performance at high rates (40 A g^−1^ and 100 A g^−1^) for ultrathin ZVO/G. (e) A photograph of a Zn‖ultrathin ZVO/G pouch cell featuring a piece of Zn anode and a piece of ultrathin ZVO/G cathode. (f) and (g) The cycling performance of a Zn‖ultrathin ZVO/G pouch cell at currents of 100 mA and 250 mA.

To evaluate the electrochemical performance, ultrathin ZVO/G and bulk ZVO were tested as cathodes in conventional coin-type AZIBs, using Zn metal anodes and 2 M Zn(OTf)_2_ aqueous electrolyte. A control sample, ZVO/G-BM, prepared by the simple ball-milling of bulk ZVO with graphene, was also included to decouple and assess the effects of graphene. The ultrathin ZVO/G cathode exhibits excellent rate capability (Fig. 2a), delivering discharge capacities of 450, 437, 429, 421, 402, 379, 361, and 346 mAh g^−1^ at progressively increased current densities of 1, 4, 7, 10, 20, 30, 40, and 50 A g^−1^, respectively. Even when the current density is increased fifty-fold from 1 to 50 A g^−1^, it retains ∼77% of its capacity (346 mAh g^−1^*vs.* 450 mAh g^−1^), indicating minimal capacity degradation under ultrahigh-rate conditions (Fig. 2a, S21 and S22). In comparison, ZVO/G-BM and bulk ZVO retain just ∼35% and ∼25%, respectively, over the same range. Moreover, the rate capacity of ultrathin ZVO/G also outperforms those of previously reported AZIB cathode materials (Fig. S23). Notably, the ZVO/G-BM electrode exhibits only comparable electrochemical performance to bulk ZVO, despite the presence of conductive graphene, confirming that simple graphene incorporation mainly contributes to electronic conductivity enhancement but cannot fundamentally address the intrinsic limitations of bulk ZVO. In contrast, the ultrathin ZVO/G heterostructure enables synergistic regulation of ion diffusion, electron transport, and interfacial charge-transfer processes through the combination of few-layer ZVO nanosheets and graphene, resulting in substantially enhanced electrochemical kinetics and superior high-rate performance.

A Ragone plot (Fig. 2b and Table S2) further highlights its superior energy and power performance. This comparison summarizes energy density *versus* power density (based on the mass of active cathode material) for the ultrathin ZVO/G cell and other representative AZIB cathodes.^32–39^ AZIBs using the ultrathin ZVO/G cathode deliver the highest reported values: 347 Wh kg^−1^/771 W kg^−1^ for energy/power density at 1 A g^−1^ (discharge time: 27 minutes) and 239 Wh kg^−1^/34 416 W kg^−1^ for energy/power density at an ultrahigh current density of 50 A g^−1^ (discharge time: 25 s). These values far exceed those of bulk ZVO-based AZIBs, which show only 227 Wh kg^−1^/761 W kg^−1^ for energy/power density at 1 A g^−1^ and 36 Wh kg^−1^/23 376 W kg^−1^ for energy/power density at 50 A g^−1^.

The cycling stability of the ultrathin ZVO/G cathode is equally impressive (Fig. 2c and d). At 0.3 A g^−1^, ultrathin ZVO/G retains 89% of its initial capacity after 150 cycles, substantially outperforming both bulk ZVO and ZVO/G-BM (Fig. 2c and S21b). At 40 A g^−1^, the ultrathin ZVO/G cathode delivers capacity retention of 69% after 30 000 cycles. Remarkably, even at an ultrahigh current density of 100 A g^−1^, an ultrathin ZVO/G-based coin cell achieves a capacity of 247 mAh g^−1^ and capacity retention of 77% after 30 000 cycles, whereas the bulk ZVO cell experiences rapid failure accompanied by a severe voltage drop. The enhanced performance is attributed to the synergistic effects of accelerated ion diffusion within the ultrathin ZVO nanosheets and the robust graphene-supported heterostructure, which together promote efficient utilization of active sites, facilitate rapid charge transfer, and maintain structural integrity during prolonged cycling.

To assess its practical viability, a Zn‖ZVO/G pouch cell with a 45 cm^2^ electrode area and ∼4.2 mg cm^−2^ cathode mass loading was assembled (Fig. 2e). The ultrathin ZVO/G-based pouch cell delivered a capacity of 43 mA h with 81% capacity retention after 500 cycles (Fig. 2f and S24a) at a discharge current of 100 mA. Even at a higher current of 250 mA, it provided 33.7 mAh with impressive 98% capacity retention after 550 cycles (Fig. 2g and S24b), demonstrating excellent cycling stability and practical viability. Notably, the failure of the cells is primarily due to internal short circuits originating from the Zn anode due to dendrite penetration (Fig. S25), rather than the degradation of the cathode. The excellent cycling stability of the ZVO/G composite cathode is attributed to its high static stability when immersed in aqueous electrolyte (Fig. S26).

### H^+^/Zn^2+^ co-insertion mechanism in ultrathin ZVO/G

**Fig. 3:**
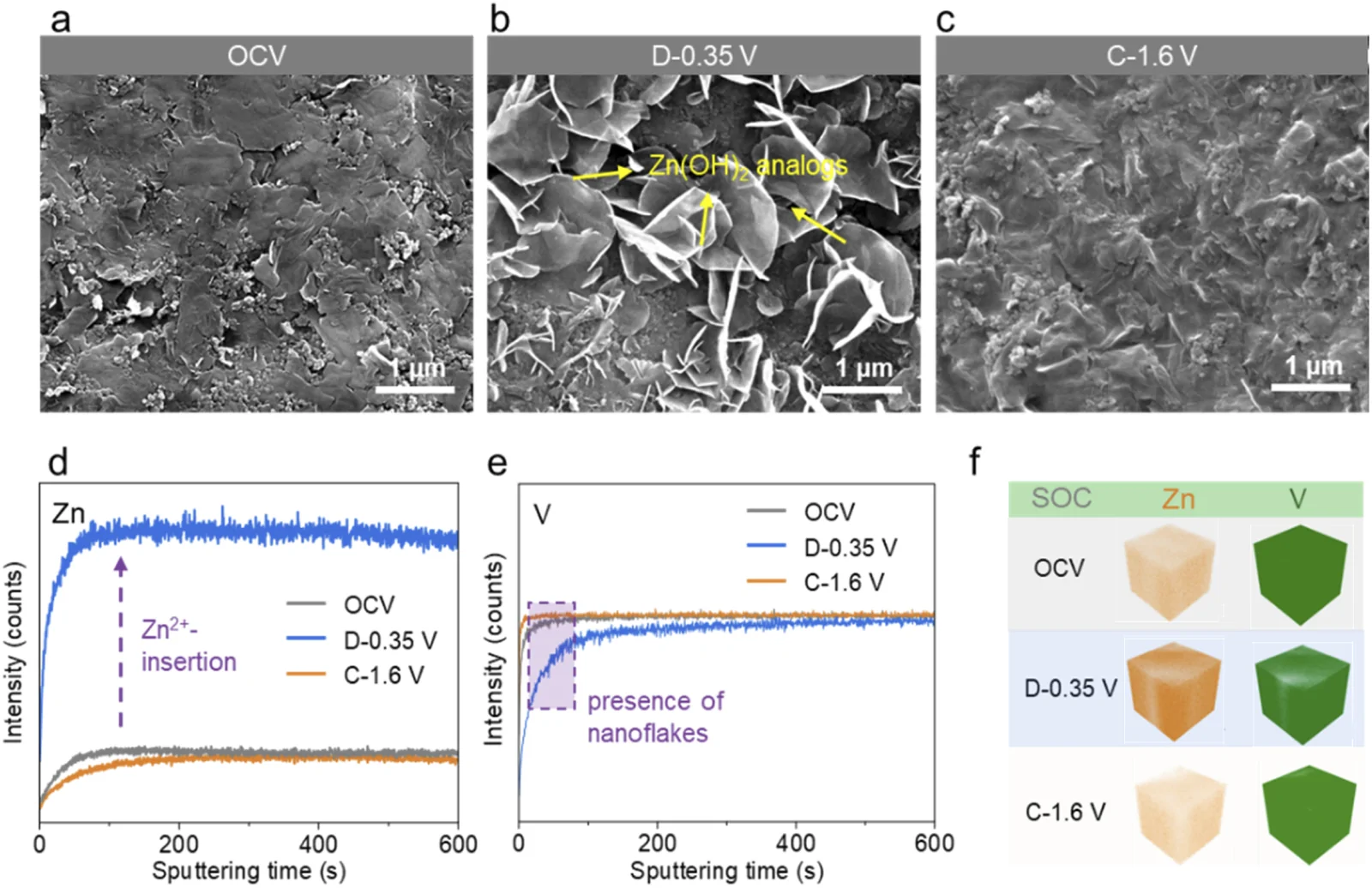
Evidence of H^+^/Zn^2+^ co-insertion in ultrathin ZVO/G cathodes. (a–c) SEM images of an ultrathin ZVO/G cathode in different charge states (a): at the open-circuit voltage, (b): fully discharged to 0.35 V, and (c): fully charged to 1.6 V). (d and e) TOF-SIMS depth profiles of Zn and V ions in ultrathin ZVO/G cathodes in various charge states. (f) Three-dimensional views of the TOF-SIMS sputtered volumes corresponding to the depth profiles (analysis area: 100 × 100 µm^2^ and ∼10 µm depth after 600 s of sputtering).

To understand the energy storage mechanism, the ultrathin ZVO/G cathode was analyzed in various charge states: at the open-circuit voltage (OCV), fully discharged to 0.35 V (D-0.35 V), and fully charged to 1.6 V (C-1.6 V). XRD patterns (Fig. S27) reveal no emergence of new crystalline phases during cycling, suggesting the absence of significant structural transformation. However, SEM images (Fig. 3a–c) show the reversible formation of nanoscale flake-like deposits on the cathode surface during discharge, which vanish upon charging, reflecting the involvement of H^+^ during cycling. Elemental analysis (Fig. S28–S30) identified these discharge-induced nanoflakes as layered Zn(OH)_2_ analogues, formed due to the localized pH increase and hydroxide precipitation during H^+^ ion insertion in the cathode during discharge.^35,40^ Notably, the ultrathin ZVO/G framework maintains its original morphology and structural integrity throughout cycling, highlighting its excellent reversibility and structural stability.

To further probe the Zn^2+^ storage behavior, XPS was employed to examine the surface composition (detection depth: ∼5 nm, Fig. S31), while time-of-flight secondary ion mass spectrometry (TOF-SIMS) was used to map the Zn^2+^ distribution within the cathode bulk (detection depth: ∼10 µm, Fig. 3d–f). As shown in Fig. S31, ZVO/G in the discharged state exhibits obviously increased Zn content accompanied by an obvious decrease in the V signal. In contrast, ZVO/G in the charged state shows Zn, V and O XPS profiles that closely resemble those of pristine ZVO/G, indicating reversible Zn^2+^ intercalation/deintercalation and excellent surface reversibility during cycling. As seen in Fig. 3d and f, the gradual increase in the Zn signal intensity at early sputtering times (before ∼100 s) originates from a transition from the surface region toward the cathode interior. The significantly higher Zn intensities in the discharge state compared with the pristine and charged states provide direct evidence for the intercalation of Zn^2+^ into ZVO/G. As seen in Fig. 3e and f, the longer sputtering time required for the V signal to reach a steady state in the discharged electrode compared to the electrodes in pristine and charged states is ascribed to surface coverage by Zn(OH)_2_ analogues, which delays the exposure of underlying ZVO/G. Overall, the charge storage mechanism in ultrathin ZVO/G involves the co-intercalation of H^+^ and Zn^2+^, consistent with that of bulk vanadium-based oxides. Importantly, the exfoliation strategy enhances the electrochemical kinetics and active-site utilization without altering the intrinsic redox chemistry or the fundamental charge-storage mechanism.

### Accelerated ion kinetics in ultrathin ZVO/G

**Fig. 4:**
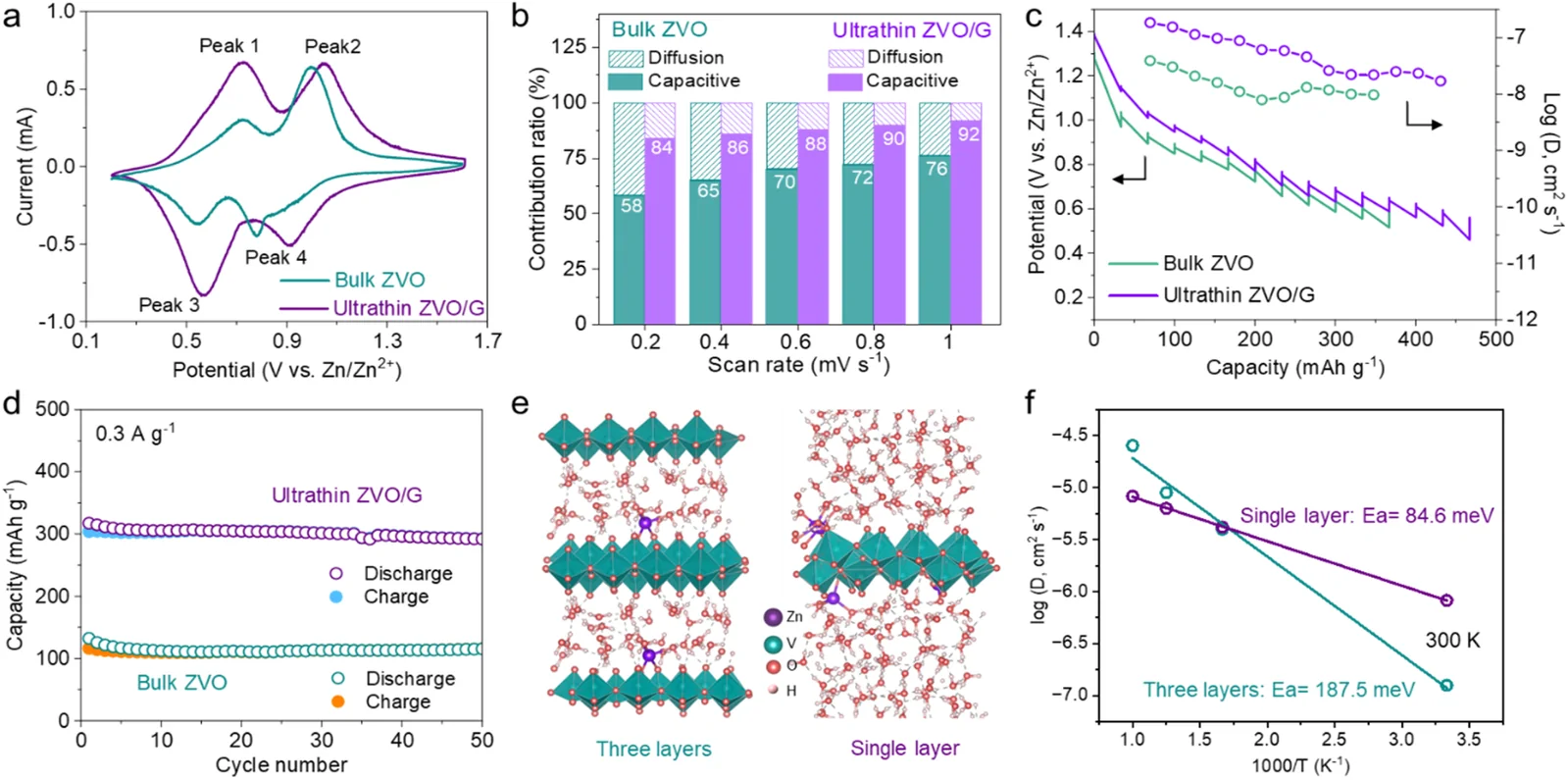
A comparison of ion storage kinetics in ultrathin ZVO/G *vs.* bulk ZVO. (a) CV curves at 0.2 mV s^−1^ for ultrathin ZVO/G and bulk ZVO. (b) Capacitive contribution ratios at various scan rates for ultrathin ZVO/G and bulk ZVO. (c) GITT-derived ion diffusion coefficients (and corresponding potentials) as a function of capacity for ultrathin ZVO/G and bulk ZVO. (d) Cycling performance at 0.3 A g^−1^ in acetonitrile-based electrolyte (1 M Zn(OTf)_2_) for ultrathin ZVO/G and bulk ZVO. (e) Structure models and (f) Arrhenius plots obtained from temperature-dependent ML-MD simulations of ZVO with three layers and one layer.

To investigate the ion transport behavior in ultrathin ZVO/G, cyclic voltammetry (CV) analysis was performed at scan rates from 0.2 to 1 mV s^−1^ for both ultrathin ZVO/G and bulk ZVO cathodes (Fig. S32 and S33). The CV curves of ultrathin ZVO/G differ markedly from those of bulk ZVO (Fig. 4a), indicating distinct redox kinetics. From the log(*i*) *vs.* log(*v*) plots, we extracted *b*-values to assess the charge-storage mechanism. For ultrathin ZVO/G, the major redox peaks show *b*-values of ∼0.91, 0.99, 0.89, and 0.96 (Fig. S32), approaching the ideal value of 1, which indicates a predominantly surface-controlled pseudocapacitive process.^41^ In contrast, bulk ZVO exhibits lower *b*-values ranging from 0.65 to 0.84 (Fig. S33), reflecting stronger diffusion-controlled intercalation behavior. Consistently, quantitative analysis of the capacitive contribution shows that ultrathin ZVO/G exhibits a surface-controlled charge-storage contribution of 84–92%, significantly higher than the range of 58–76% observed for bulk ZVO (Fig. 4b). In addition, to distinguish the faradaic pseudocapacitive contribution from the non-faradaic electrical double-layer capacitance, the CV currents were background-calibrated,^42^ as presented in Fig. S34 and S35. After excluding non-faradaic contributions, the calculated faradaic pseudocapacitive contributions are slightly lower than the total pseudocapacitive contributions obtained without background correction for both bulk ZVO and ultrathin ZVO/G. Nevertheless, compared with bulk ZVO (49.2–63.8%), ultrathin ZVO/G also exhibits a noticeably higher intrinsic pseudocapacitive contribution (69.0–77.1%). This enhanced pseudocapacitive behavior in ultrathin ZVO/G is attributed to the ultrathin ZVO nanosheet architecture and intimate ZVO/graphene interface, which expose abundant active sites, shorten ion diffusion pathways, and facilitate rapid interfacial charge transfer.

To further probe the Zn^2+^ diffusion kinetics, the galvanostatic intermittent titration technique (GITT) was employed. As shown in Fig. 4c, the ion diffusion coefficient in ultrathin ZVO/G is nearly eight times higher than that in bulk ZVO, demonstrating substantially accelerated ion migration within the exfoliated nanosheet architecture. To isolate Zn^2+^ transport from H^+^ effects, the electrochemical performance of ZVO/G was also studied in an aprotic organic electrolyte (1 M Zn(OTf)_2_ in acetonitrile). Even without H^+^, ultrathin ZVO/G delivered a high reversible capacity of ∼305 mAh g^−1^, significantly outperforming the value of only ∼120 mAh g^−1^ for bulk ZVO (Fig. 4d), providing direct evidence for significantly faster Zn^2+^ diffusion in ultrathin ZVO/G compared to bulk ZVO.

Machine-learning-driven molecular dynamics (ML-MD) simulations further indicate the critical role of layer thinning in accelerating Zn^2+^ transport. A single-layer ZVO model exhibits a significantly higher Zn^2+^ diffusion coefficient at room temperature (8.24 × 10^−7^ cm^2^ s^−1^) than a three-layer ZVO model (1.26 × 10^−7^ cm^2^ s^−1^). Meanwhile, the corresponding Zn^2+^ diffusion barrier is significantly reduced from 187.5 meV in three-layer ZVO to 84.6 meV in single-layer ZVO. These results demonstrate that reducing the number of ZVO layers effectively facilitates ion migration and lowers the energetic barrier for Zn^2+^ diffusion, thereby enabling faster charge-storage kinetics and ultrafast charging capabilities. In good agreement with the experimental observations, the simulations confirm that the enhanced ion transport originates from weakened interlayer interactions and the improved accessibility of interlayer Zn^2+^ migration pathways in ultrathin ZVO structures.

These findings highlight that exfoliating bulk ZVO into atomically thin nanosheets, coupled with conductive graphene integration, dramatically enhances ion transport and maximizes active-site utilization. The ultrathin ZVO/G heterostructure thus enables rapid charge storage, high rate-capability and excellent cycling stability, making it highly suitable for fast-charging AZIB applications. More broadly, this top-down exfoliation strategy provides a versatile platform for engineering ultrathin electrode architectures and may be extended to other layered vanadium oxides and advanced battery systems, offering a general approach for optimizing ion/electron transport in energy storage materials.

## Conclusions

Vanadium-based oxide nanosheets with an ultrathin thickness of ∼2 nm were successfully produced *via* the top-down exfoliation of bulk ZVO, providing an effective strategy for accelerating ion transport and enabling fast-charging AZIBs. This top-down exfoliation process significantly increases the exposure of accessible redox-active sites while weakening interlayer interactions, thereby facilitating rapid ion diffusion in the layered framework. The resulting ZVO nanosheets were further combined with conductive graphene *via* electrostatic assembly to construct a robust ZVO/G heterostructure. This ZVO/G heterostructure delivers a near-eightfold enhancement in the solid-state ion diffusion coefficient compared with bulk ZVO, accompanied by a substantially reduced diffusion barrier (84.6 meV *versus* 187.5 meV). Meanwhile, graphene serves as an efficient electron-conducting network, prevents nanosheet restacking, and promotes interfacial charge transfer and pseudocapacitive charge-storage behavior. Benefiting from these synergistic structural advantages, Zn‖ultrathin ZVO/G cells exhibit outstanding high-rate performance, retaining ∼77% capacity retention when the discharge rate increases from 1 to 50 A g^−1^. They also achieve high energy/power densities (*e.g.*, 239 Wh kg^−1^/34 416 W kg^−1^ at 50 A g^−1^), far surpassing bulk ZVO, which retains only ∼25% of its capacity and delivers much lower energy/power output (36 Wh kg^−1^/23 376 W kg^−1^ at 50 A g^−1^). Furthermore, pouch cells with high mass loading (∼4.2 mg cm^−2^) and large area (45 cm^2^) exhibit outstanding cycling stability, with 98% capacity retention after 550 cycles, demonstrating strong practical viability. Overall, this ultrathin cathode design provides an effective pathway toward fast-charging battery technologies and offers a generalizable strategy for developing high-rate electrode materials for next-generation energy storage systems, including Li-, Na-, K-, and Zn-based batteries.

## Author contributions

W. J. and X. N. contributed equally to this work. W. Y., K. Z., and W. J. conceived and designed this work. W. J., X. N. and K. Z. collected and analyzed the data. W. X. and Z. W assisted the material synthesis and characterization. K. Z., W. J. and X. N. wrote the article. W. Y., and K. Z. revised the article. W. Y. and K. Z. supervised the project. All authors contributed to discussion of the results.

## Conflicts of interest

There are no conflicts to declare.

## Supplementary Material

SC-OLF-D6SC05963E-s001

## Data Availability

The data that support the findings of this article are available from the corresponding author upon request. Supplementary information (SI): includes experimental details, Fig. S1–S35 and Tables S1 and S2. See DOI: https://doi.org/10.1039/d6sc05963e.
