# Supplementary material for: Atomically thin vanadium oxide nanosheets via top-down exfoliation for ultrafast-charging zinc-ion batteries
Source: Chem Sci. 2026 Sep 7. Online ahead of print. doi: 10.1039/d6sc05963e (PMC13576155; doi:10.1039/d6sc05963e)
Supplement: Supplementary file 1 [file SC-OLF-D6SC05963E-s001.pdf]

Supplementary Information (SI) for Chemical Science.
This journal is © The Royal Society of Chemistry 2026

Supplementary Information

Atomically thin vanadium oxide nanosheets via top-down exfoliation for ultrafast-charging zinc-ion batteries

Weikang Jiang[† a], Xiling Niu[† a,c], Kaiyue Zhu* [a,b], Weili Xie[a,b], Zhengsen Wang[a], Weishen Yang* [a,b]

[a] State Key Laboratory of Catalysis, Dalian Institute of Chemical Physics, Chinese Academy of Sciences, Dalian, 116023, China;

[b] University of Chinese Academy of Sciences, Beijing, 100049, China;

[c] Henan Key Laboratory of Quantum Materials and Quantum Energy, School of Quantum Information Future Technology, Henan University, Kaifeng 475001 (China)

E-mail: yangws@dicp.ac.cn; zky218@dicp.ac.cn

[†]These authors contribute equally to this work

This PDF file includes:

Materials and methods
Fig. S1 to S35
Table S1 and S2
References

MATERIALS AND METHODS

Chemical

NH_4VO_3, $ZnSO_4·7H_2O$ and $Zn(CF_3SO_3)_2$ were purchased from Sigma Aldrich. N-Methylpyrrolidone (99%), N-N-dimethylformamide (99%), N-N-dimethylacetamide (99%), methanol (99%), ethanol (99%), isopropanol (99%), acetonitrile (99%) and acetone (99%) were purchased from Sinopharm Chemical Reagent Co., Ltd. They were all used without further purification.

Synthesis of bulk ZnV_6O_{16}�nH_2O (ZVO)[1]

In a typical procedure, 0.234 g of commercial NH_4VO_3 powder was dissolved in 30 mL of deionized water at 70 °C followed by the addition of 0.02 g chitosan under continuous magnetic stirring at room temperature for 1 h. Subsequently, 5 mL of H_2O_2 (35wt%) was added to the above mixed solution and stirred for 20 min. Afterwards, a certain amount of nitric acid was added dropwise to adjust the pH of the solution to about 2, followed by further stirring for 30 min, resulting in an orange solution. After that, the mixture was transferred into a 100 mL Teflon-lined sealed autoclave and maintained at 160°C for 18 h. After cooling down to room temperature naturally, the solid product was collected by filtration, washed three times with deionized water and absolute ethanol, and dried in air at 60 °C for 12 h, yielding orange powders. Next, 0.2g of orange powders were dispersed in 50 mL of $ZnSO_4$ (2 M) solution and stirred magnetically at room temperature for 72 h. The resulting dark red precipitate was collected and washed with distilled water 3 times. Finally, ZnV_6O_{16}�nH_2O was obtained after drying above dark red precipitate at 60 °C for 12 h.

Exfoliation of bulk ZVO to atomically thin ZVO nanosheets

0.02 g of bulk $ZnV_6O_{16}·nH_2O$ precursor was dispersed in 100 mL of N-Methylpyrrolidone (NMP) solvent and sealed in a 150 mL ball milling jar. The jar was mounted on a planetary ball mill and the mixture was wet milled at a steady speed of 60 rpm for 6 hours, with reverse rotation every 15 seconds. Following ball milling, the dispersion underwent further exfoliation via ultrasonic treatment in a water bath sonicator for 2 hours. After ultrasonication, the suspension was centrifuged at a relatively low speed of 2000 rpm to remove unexfoliated particles. The supernatant, containing a stable ZVO colloidal suspension, was obtained. ZVO nanosheets were then obtained by freeze-drying the colloidal suspension to remove the solvent. For comparison, similar exfoliation processes were also performed in solvents beyond NMP (such as dimethyl formamide, dimethylacetamide, acetonitrile, acetone, methanol, ethanol, and isopropyl).

Preparation of ultrathin ZVO/G nanosheets

A mixture of 0.0857 g of ZVO nanosheets and 0.0143 g of graphene powder was dispersed in 100 mL of NMP solvent via ultrasonication for 30 min to achieve uniform dispersion. The resulting suspension was then transferred into a 200 mL Teflon-lined stainless steel autoclave and subjected to solvothermal treatment at 180°C for 12 h. The solvothermal process not only facilitated further exfoliation of the ZVO and graphene, but also promoted their assembly into ultrathin ZVO/graphene (ZVO/G) heterostructures via electrostatic interactions. After cooling naturally to room temperature, the product was collected by filtration using a nylon filter membrane with a pore size of 50 nm. The filtered material was then freeze-dried to remove the solvent, yielding the ultrathin ZVO/G heterostructures.

Cathode preparation, battery assembly and electrochemical

Electrochemical tests were carried out on CR2032-type coin cells and pouch cells. To prepare a cathode, 60 wt% active materials, 25 wt% Super-P, and 15 wt% polyvinylidene fluoride (PVDF) were thoroughly mixed and dispersed into N-Methyl pyrrolidone (NMP). For the cathodes in CR2032-type coin cells, the resultant slurry was then coated uniformly onto a 14 mm diameter stainless steel mesh (SSM), achieving an active material loading of approximately 1 mg cm^{-2}. For the cathodes in pouch cells, the resultant slurry was then coated uniformly onto a stainless steel foil, achieving an active material loading of approximately 4.2 mg cm^{-2}. The coated electrodes were dried at 60 ºC for 12 h.

In a full ZIB cell, zinc foil was used as the anode, 2 M $Zn(OTf)_2$ as the electrolyte, and glass microfiber filters as the separator. The CR2032-type coin cells and pouch cells were assembled in air and tested using a LAND battery testing system (CT2001A) within a potential window of 0.35-1.6 V (vs Zn/Zn^{2+}).

Materials characterizations

X-ray diffraction (XRD) was performed on a X'pert Pro-1 X-ray diffractometer with a Cu Kα1 X-ray source (λ = 1.5405 Å) to determine the phase structures. The data were recorded from 5º to 60º with an interval of 0.02º and a scan speed of 5º min^{-1}. The morphologies of the samples were captured with a scanning electron microscopy (SEM, Quanta 200F). Microscopic morphologies and elemental mapping were obtained by a Field-emission scanning electron microscopy (FESEM, JSM-7900F) with a Max80 energy dispersive spectroscopy (EDS). The crystalline structures and morphologies of the samples were also acquired with a Transmission electron microscopy (TEM, HT7700) and high-resolution transmission electron microscopy (HRTEM, Titan Themis ETEM G3). The thickness of samples was analyzed by atomic force microscopy (AFM, JPK NanoWizard). X-ray photoelectron spectroscopy (XPS, Thermofisher Escalab 250 Xi+) and Raman spectroscopy (NanoWizard) were used to record more

information about the chemical bonding conditions. Time-of-flight secondary ion mass spectrometry (TOF-SIMS, IONTOF GmbH) is used to detect elemental composition and depth distribution under different charge and discharge conditions. Gas adsorption measurements were performed using a Micromeritics ASAP 2020 plus physisorption analyzer. The specific surface area was determined from the Nitrogen adsorption-desorption isotherms collected at 77 K using the Brunner-Emmet-Teller method. UV-vis spectrophotometry was conducted on a Shimadzu UV-1900 spectrophotometer. Inductively coupled plasma optical emission spectrometer (PerkinElmer ICP-OES 7300DV) was used to analyze the concentration of elements. Thermogravimetric analysis (TGA) was performed using a Pyris Diamond TG/DTA thermal analyzer.

Computational Methods.

The Zn^{2+} diffusion coefficients were calculated using molecular dynamics (MD) simulations based on a machine learning interatomic potential (ML-IAP). The interatomic potential was constructed using the DPA-3 architecture, derived from the DPA-3.2-5M foundation model, which enables near-first-principles accuracy at substantially reduced computational cost[2]. All MD simulations were carried out using the Atomic Simulation Environment (ASE)[3]. A Langevin thermostat with a friction coefficient of 0.01 fs^{-1} was employed to maintain the target temperatures. A time step of 0.5 fs was adopted for all simulations, with each run consisting of a 1 ps equilibration stage (2,000 steps) followed by a 10 ps production stage (20,000 steps). Atomic trajectories were saved every 10 steps for subsequent analysis. The diffusion coefficients were extracted from the linear regime of the mean square displacement (MSD) using the Einstein relation. The activation barriers for ion diffusion were obtained by fitting the temperature-dependent diffusion coefficients to the Arrhenius equation. The linearity of the MSD was carefully verified to ensure that the diffusive regime was adequately sampled across all temperatures.

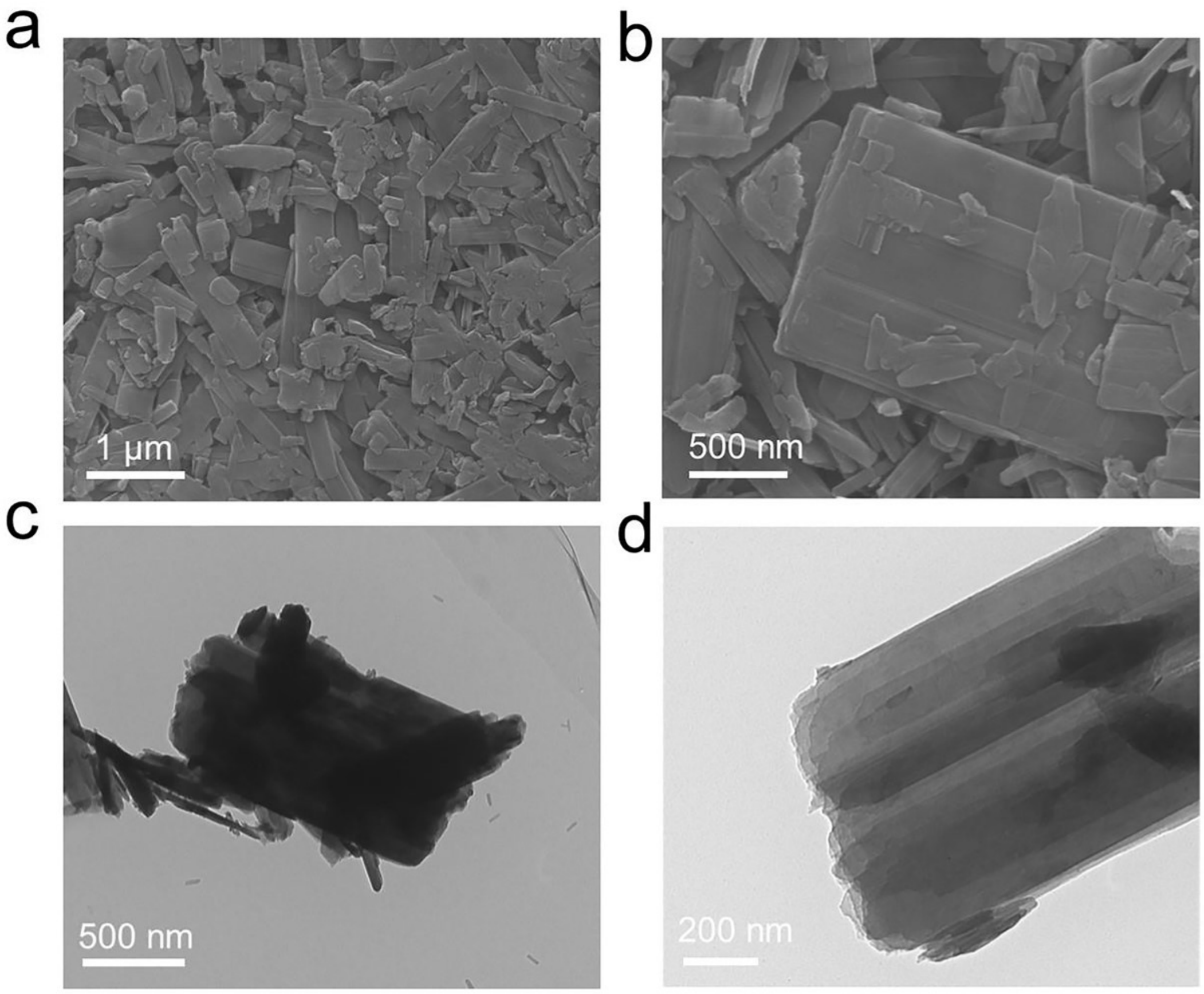

Fig. S1. (a), (b) Scanning electron microscopy (SEM) and (c), (d) Transmission electron microscopy (TEM) images of bulk ZVO.

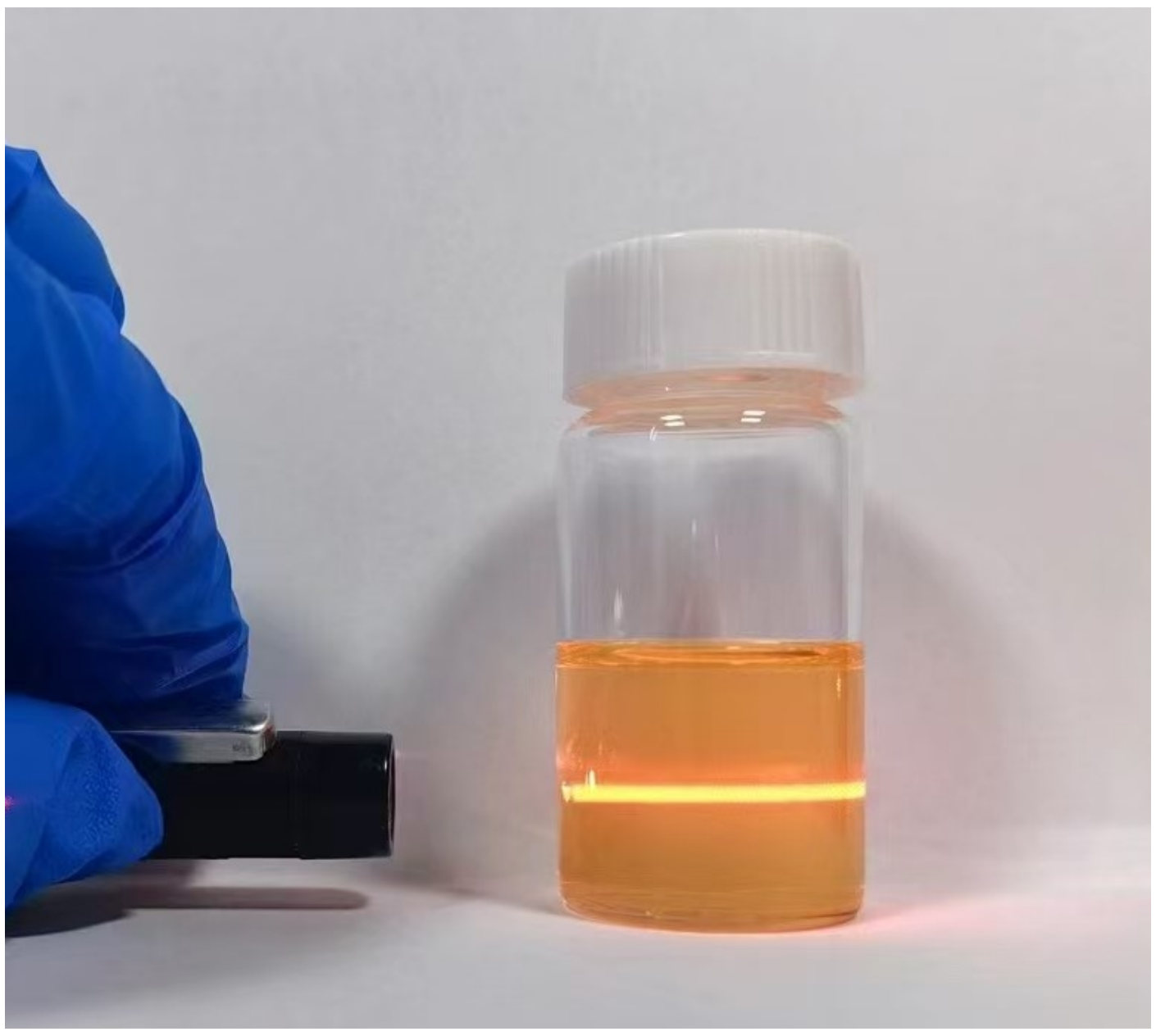

Fig. S2. Tyndall effect of an ultrathin ZVO suspension in NMP.

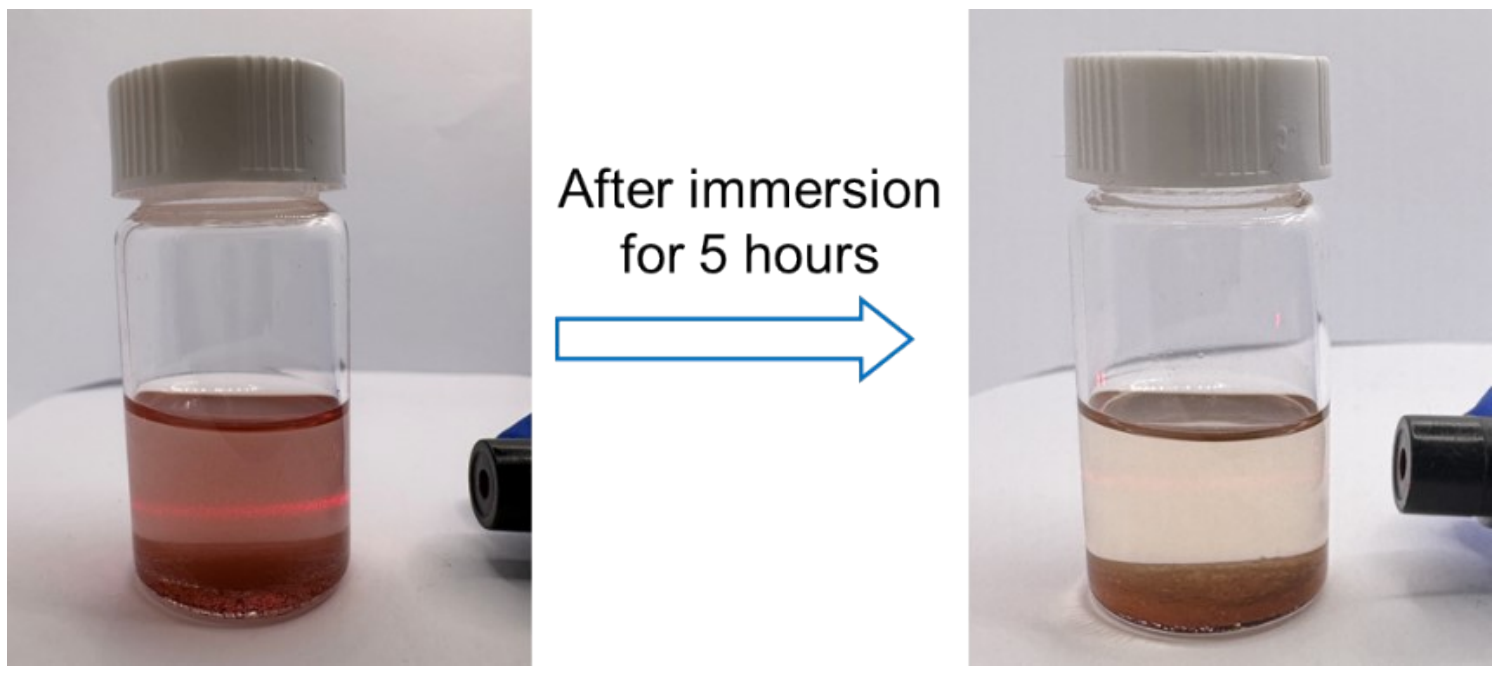

Fig. S3. Optical images of bulk ZVO (0.02 g) dispersed in NMP solution (10 mL) and after immersion for 5 hours.

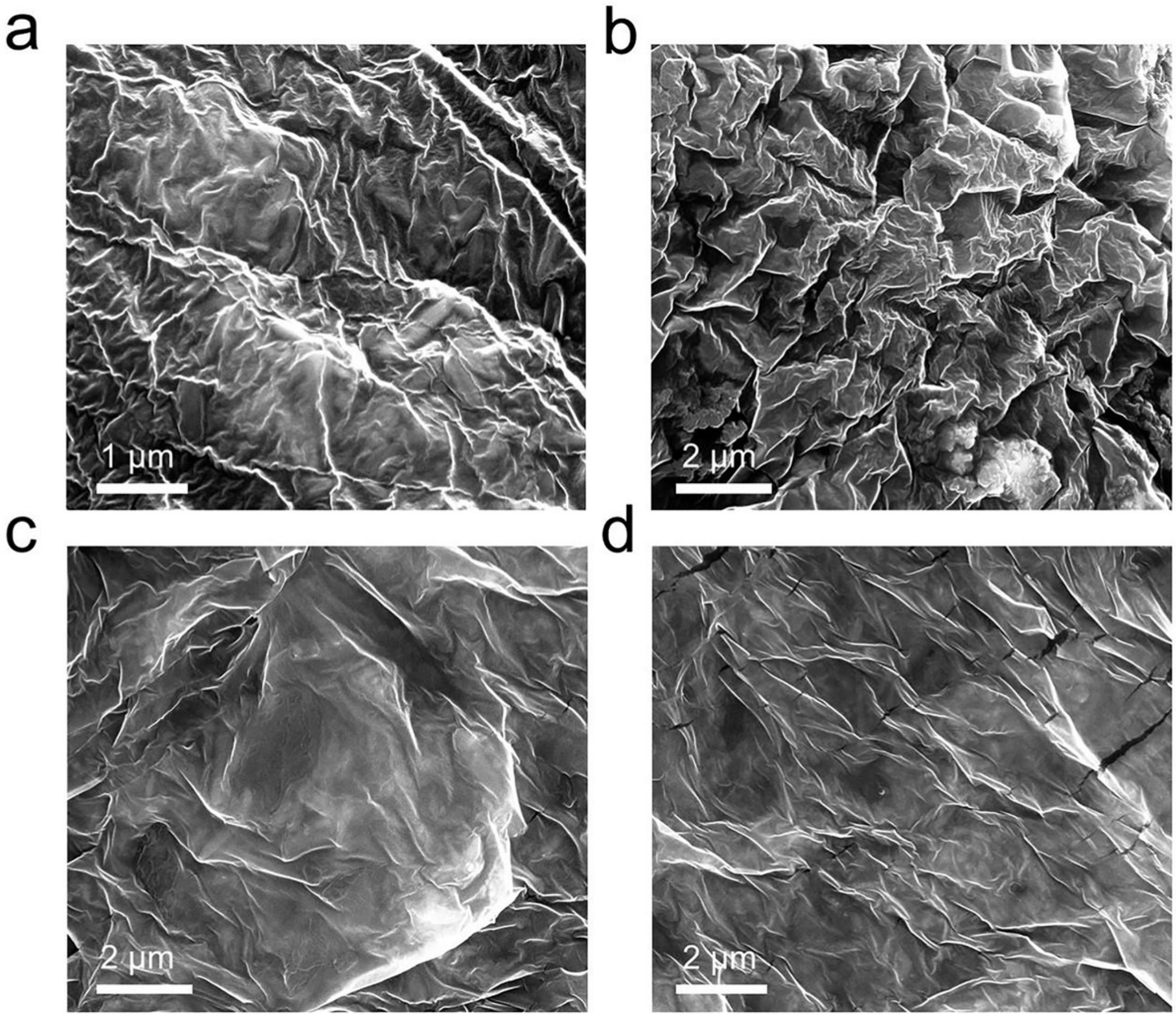

Fig. S4. SEM images of ultrathin ZVO nanosheets.

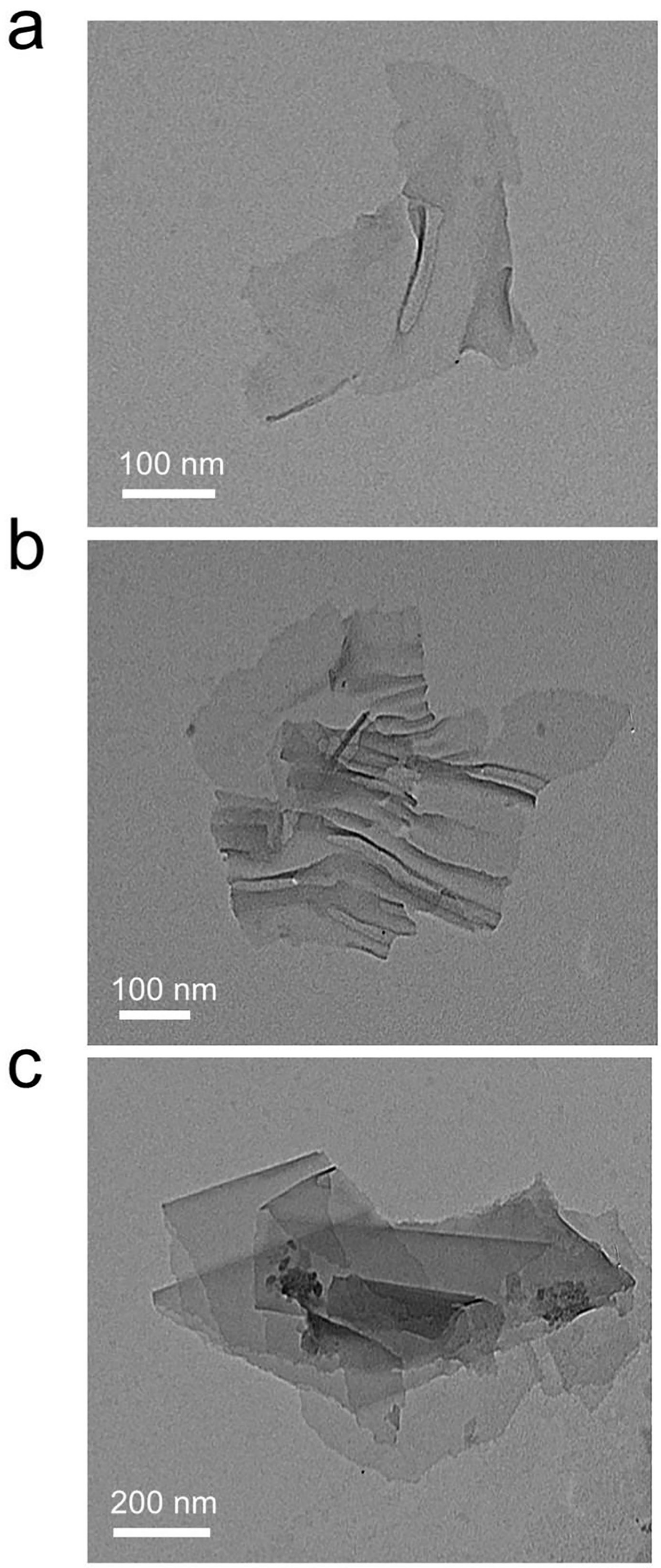

Fig. S5. TEM images of ultrathin ZVO nanosheets.

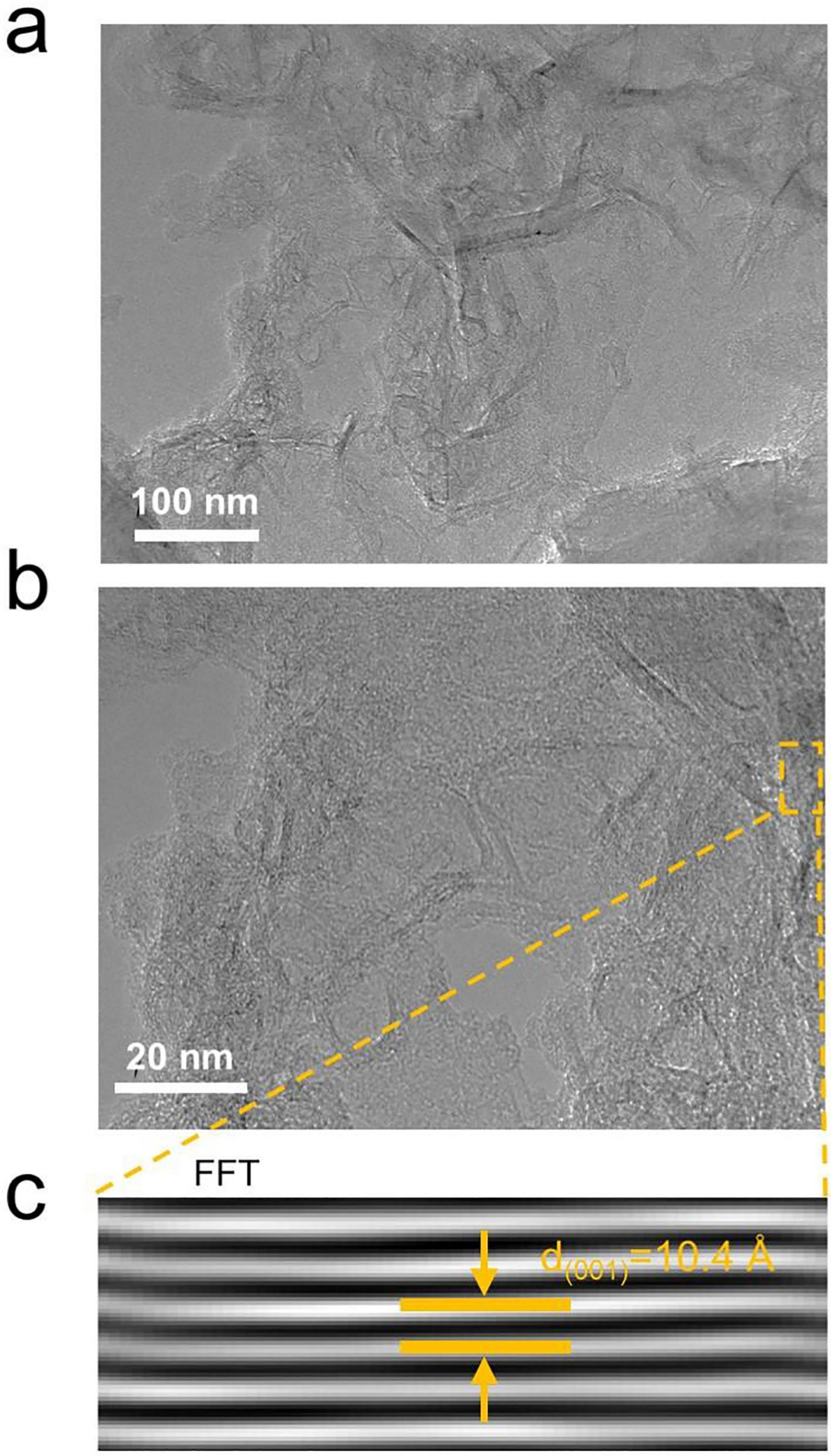

Fig. S6. (a), (b) TEM images of ultrathin ZVO nanosheets and (c) Fast Fourier Transform (FFT) of orange rectangle in Fig. S5b.

The lattice spacing of 10.4 Å in Fig. S5c corresponds well with the (001) plane of ZVO, confirming the nanosheets are composed of ZVO.

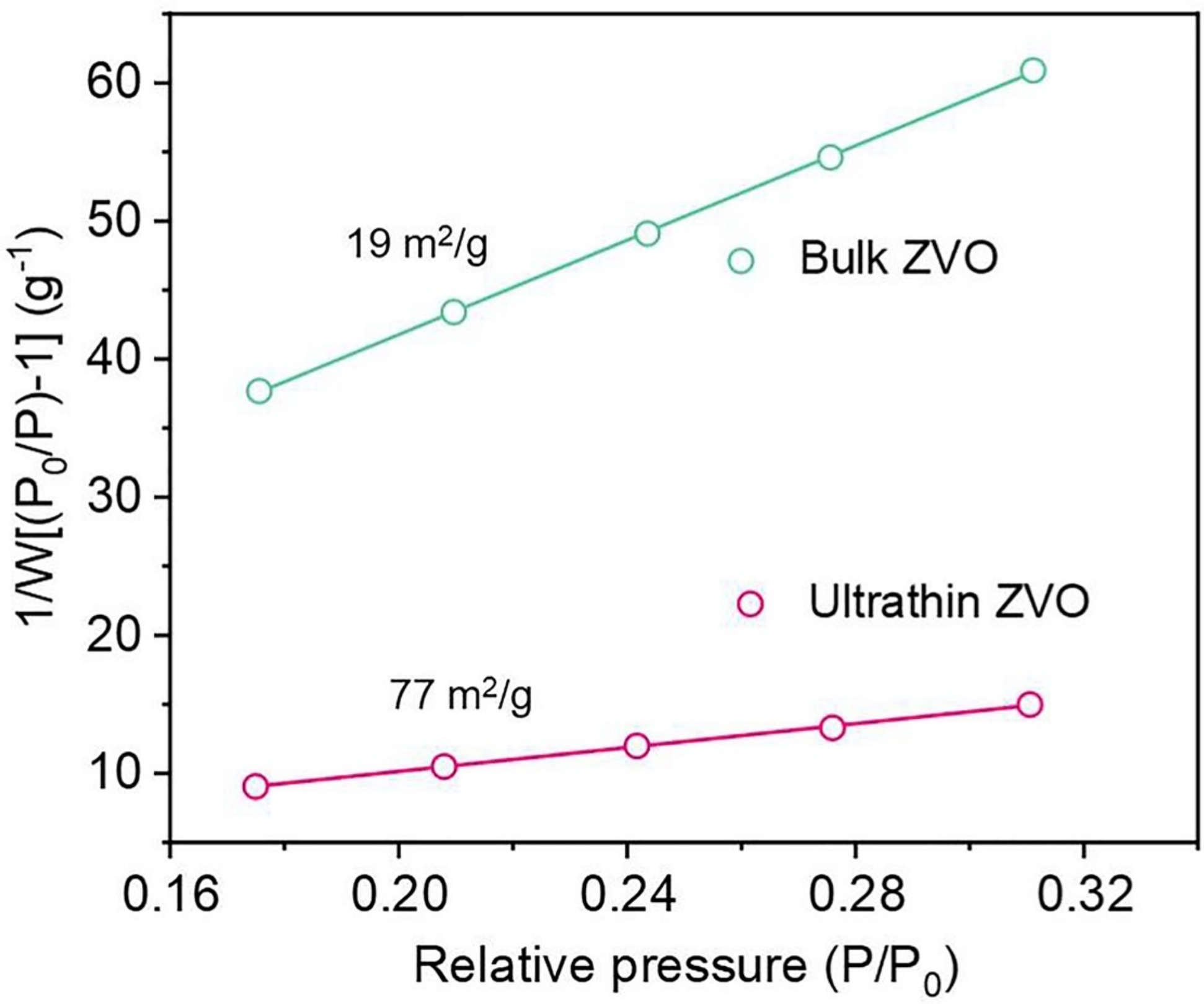

Fig. S7. Nitrogen adsorption-desorption isotherms of bulk ZVO and ultrathin ZVO nanosheets.

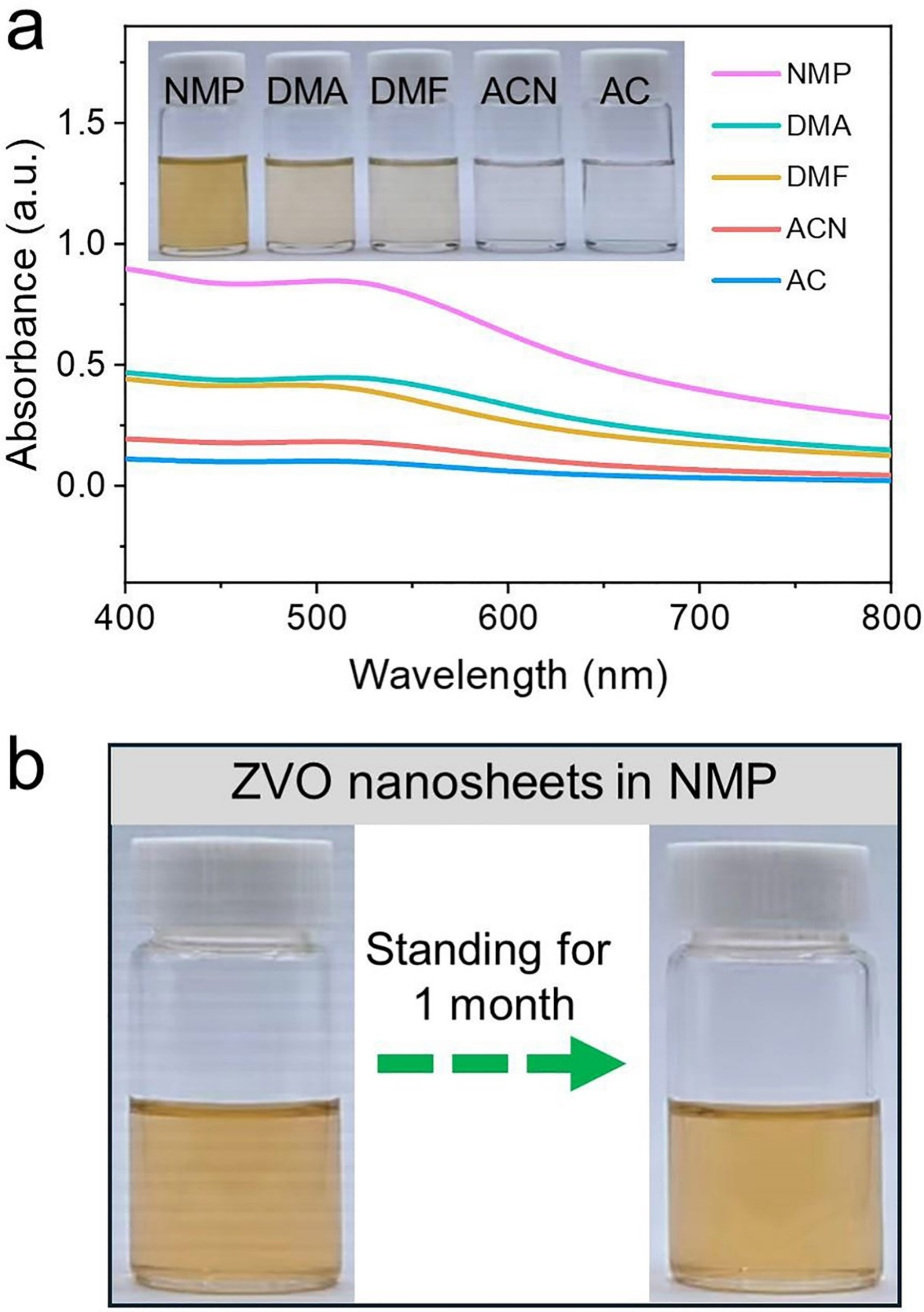

Fig. S8. (a) UV-visible spectra of ZVO nanosheets after exfoliation in different solvents of NMP, dimethyl formamide (DMF), dimethylacetamide (DMA), acetonitrile (ACN), acetone (AC). (b) Photographs of ZVO nanosheets dispersed in NMP after initial exfoliation and following 1 month of storage.

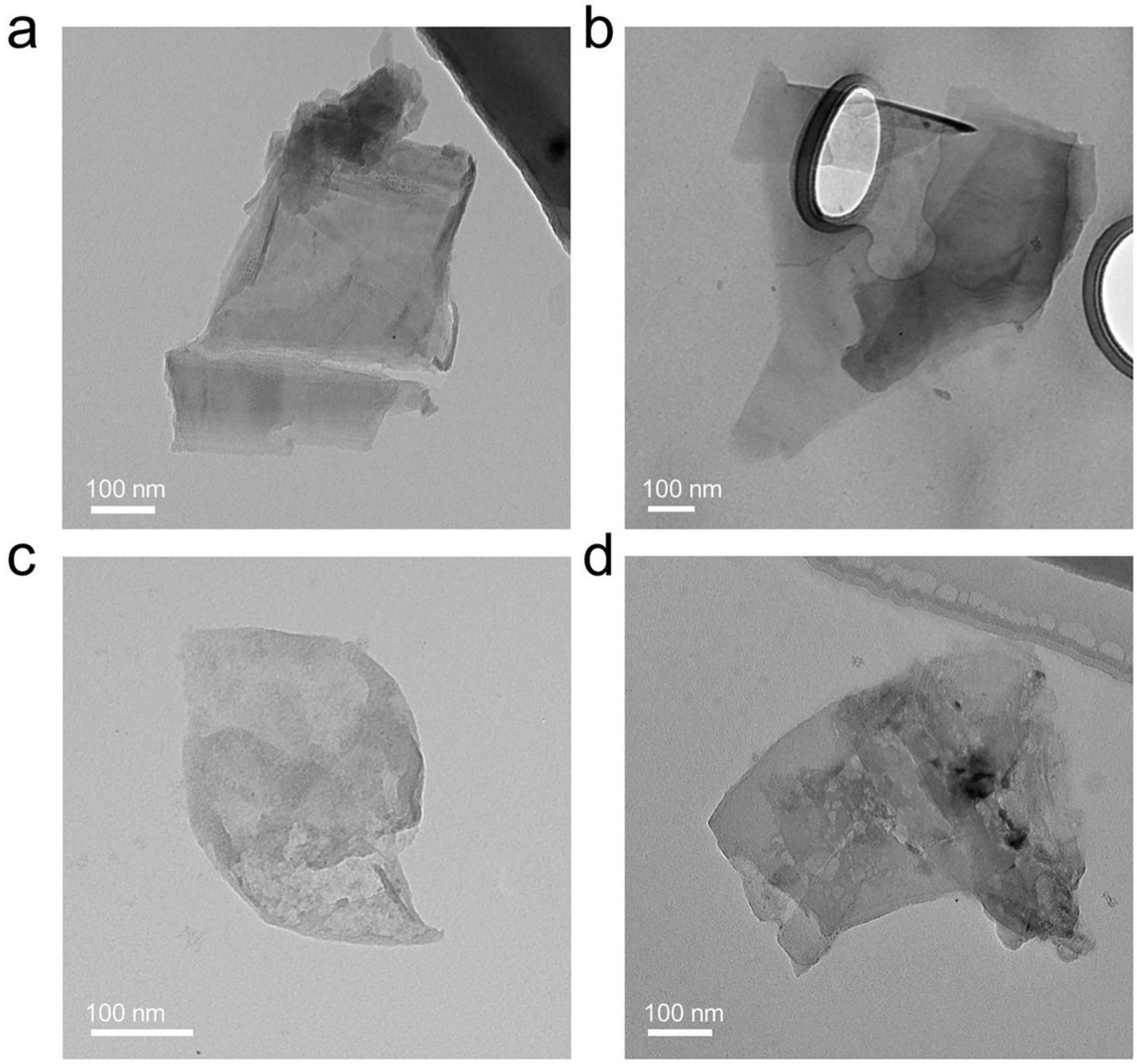

Fig. S9. TEM images of ZVO nanosheets obtained by exfoliating bulk ZVO in solvents of (a), (b) dimethyl formamide (DMF) and (c), (d) dimethylacetamide (DMA).

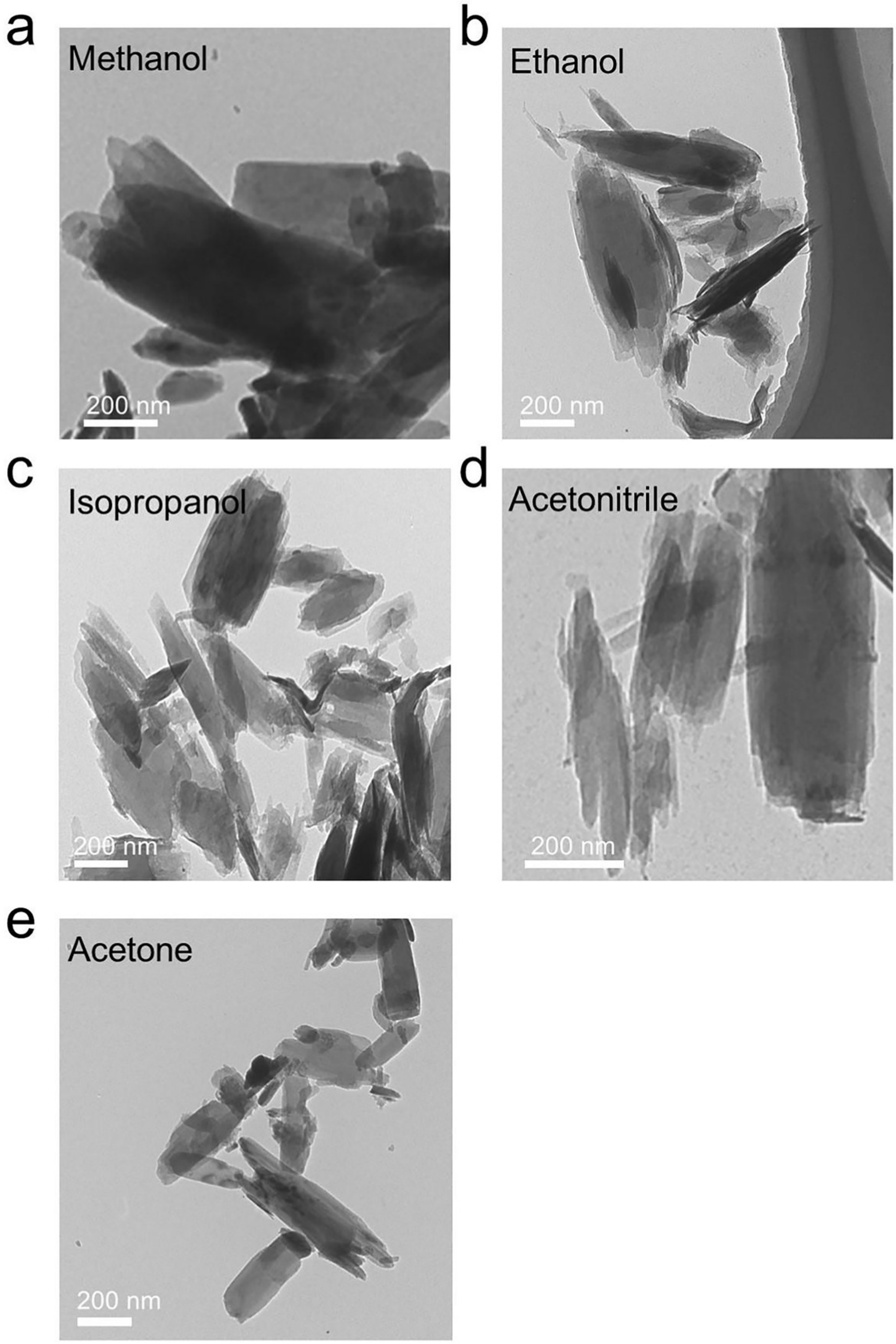

Fig. S10. TEM images of ZVO nanosheets obtained by exfoliating bulk ZVO in solvents of (a) methanol, (b) ethanol, (c) isopropanol, (d) acetonitrile, and (e) acetone.

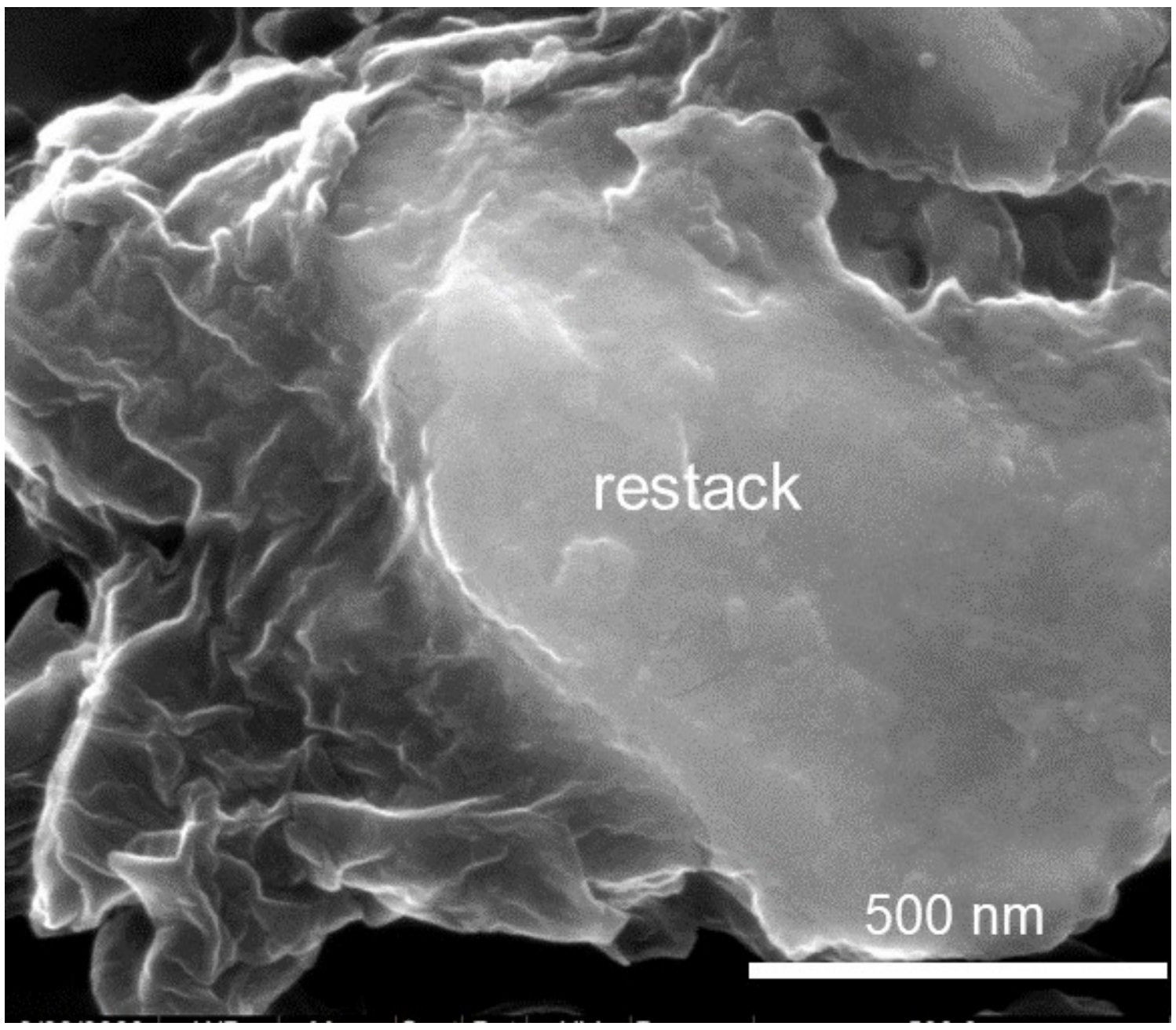

Fig. S11. SEM image of exfoliated ZVO, showing a stacking phenomenon after drying.

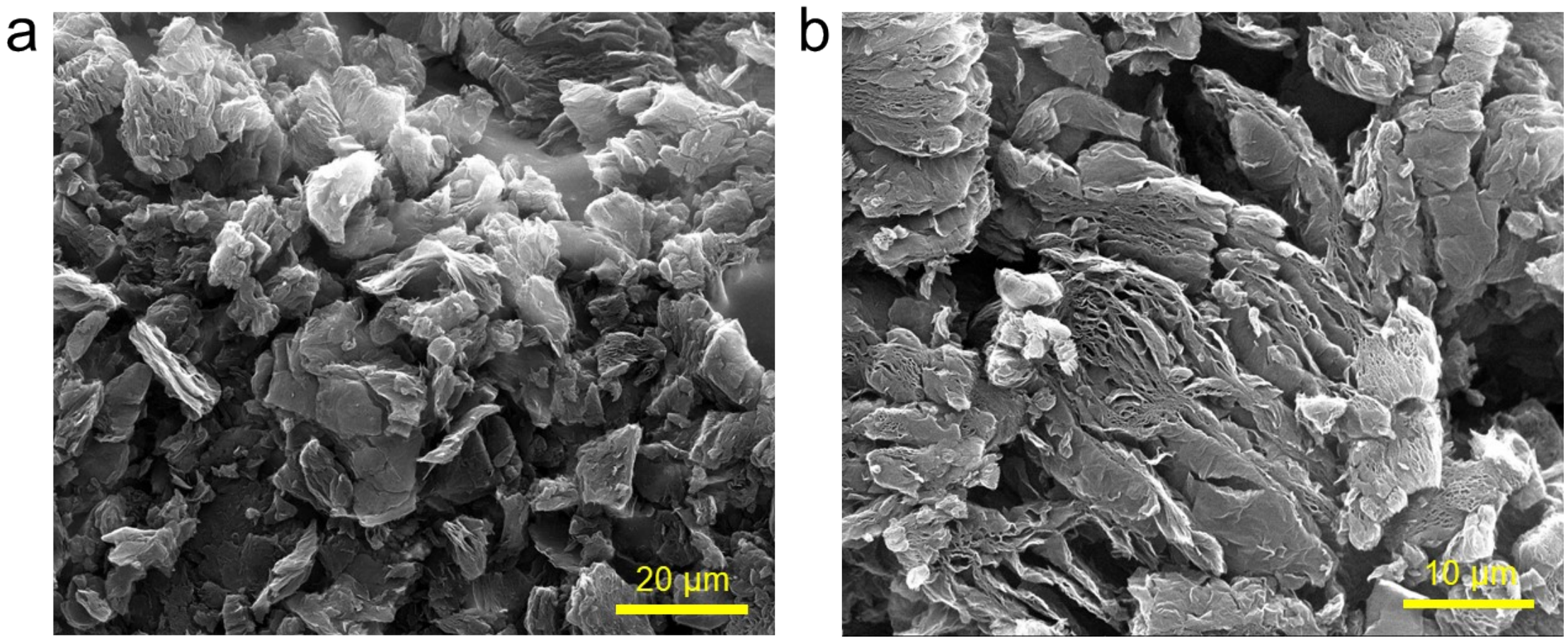

Fig. S12. SEM images of few-layer graphene.

The graphene nanosheets exhibit a morphology distinctly different from that of the ultrathin ZVO nanosheets (Fig. 1c and S4).

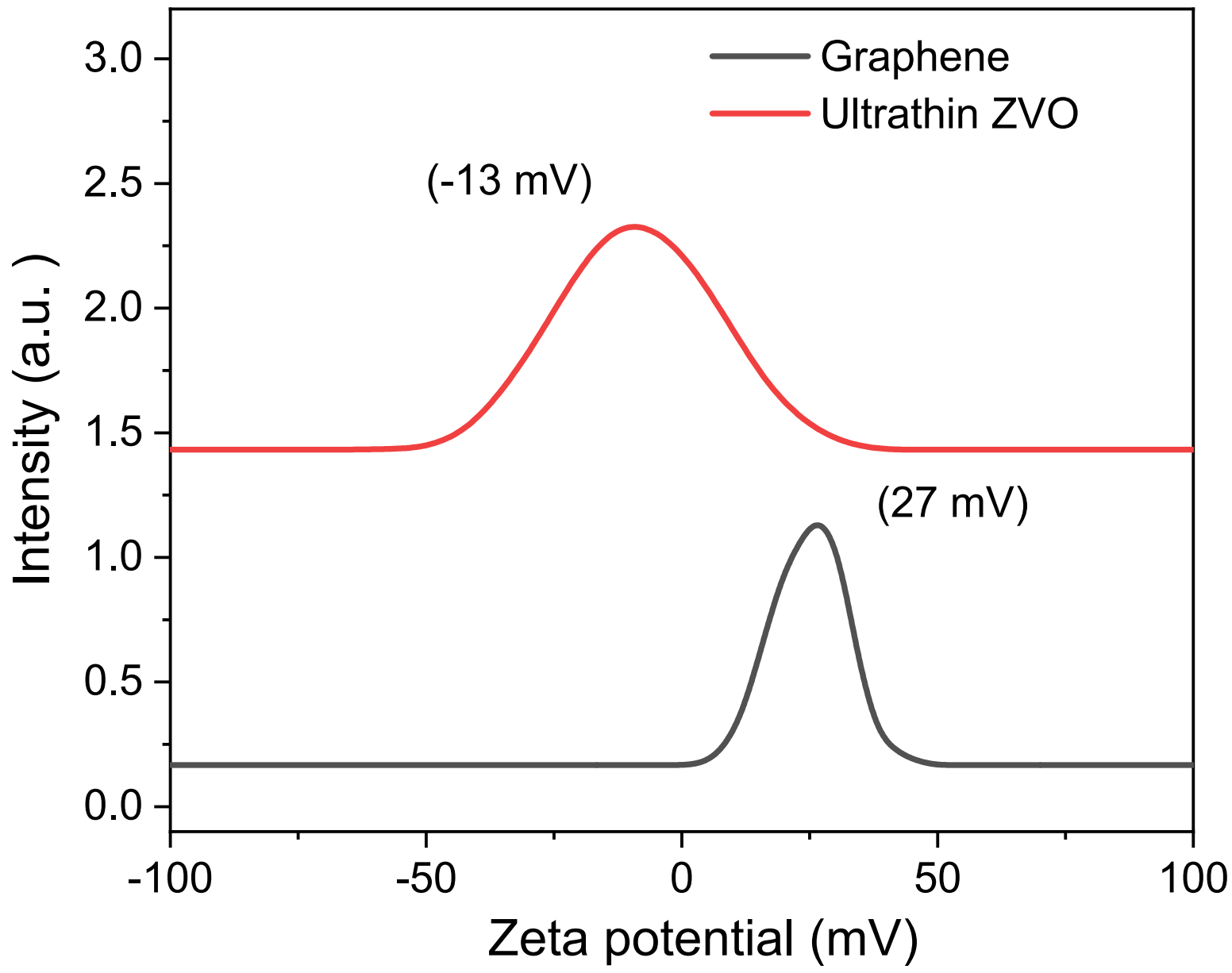

Fig. S13. Zeta potential distribution curves for colloidal suspensions of graphene and ultrathin ZVO nanosheets in NMP solvent.

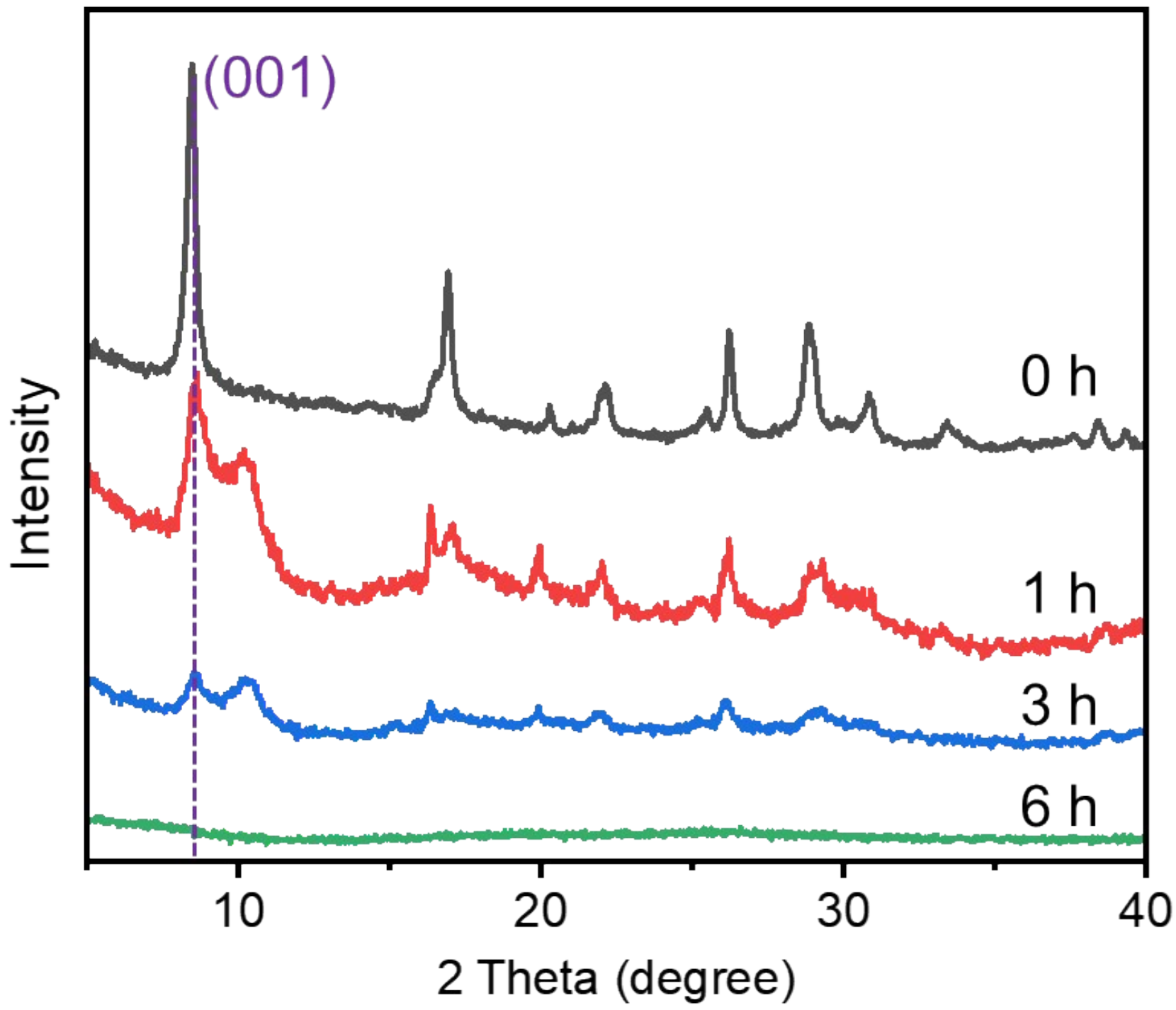

Fig. S14. XRD patterns of ZVO/G prepared with varying ball-milling durations (0 h, 1 h, 3 h, 6 h).

ZVO possesses a monoclinic structure with the space group of P2/m. The characteristic XRD peaks of ZVO are assigned to the (00l) crystal planes, which are strongly dependent on the stacking layers. In the hewettite structure, a decrease in the number of layers (i.e., the number of stacked V_6O_{16} layers along the c-axis direction), leads to a lower intensity of the diffraction peaks. This is primarily caused by the combined effects of a reduced coherent scattering volume of the crystal and weakened interlayer interference. In addition, both the reduced out-of-plane ordering due to interlayer gliding and the size/thickness effect of the ultrathin nanosheets will result in the decreased peak intensity.

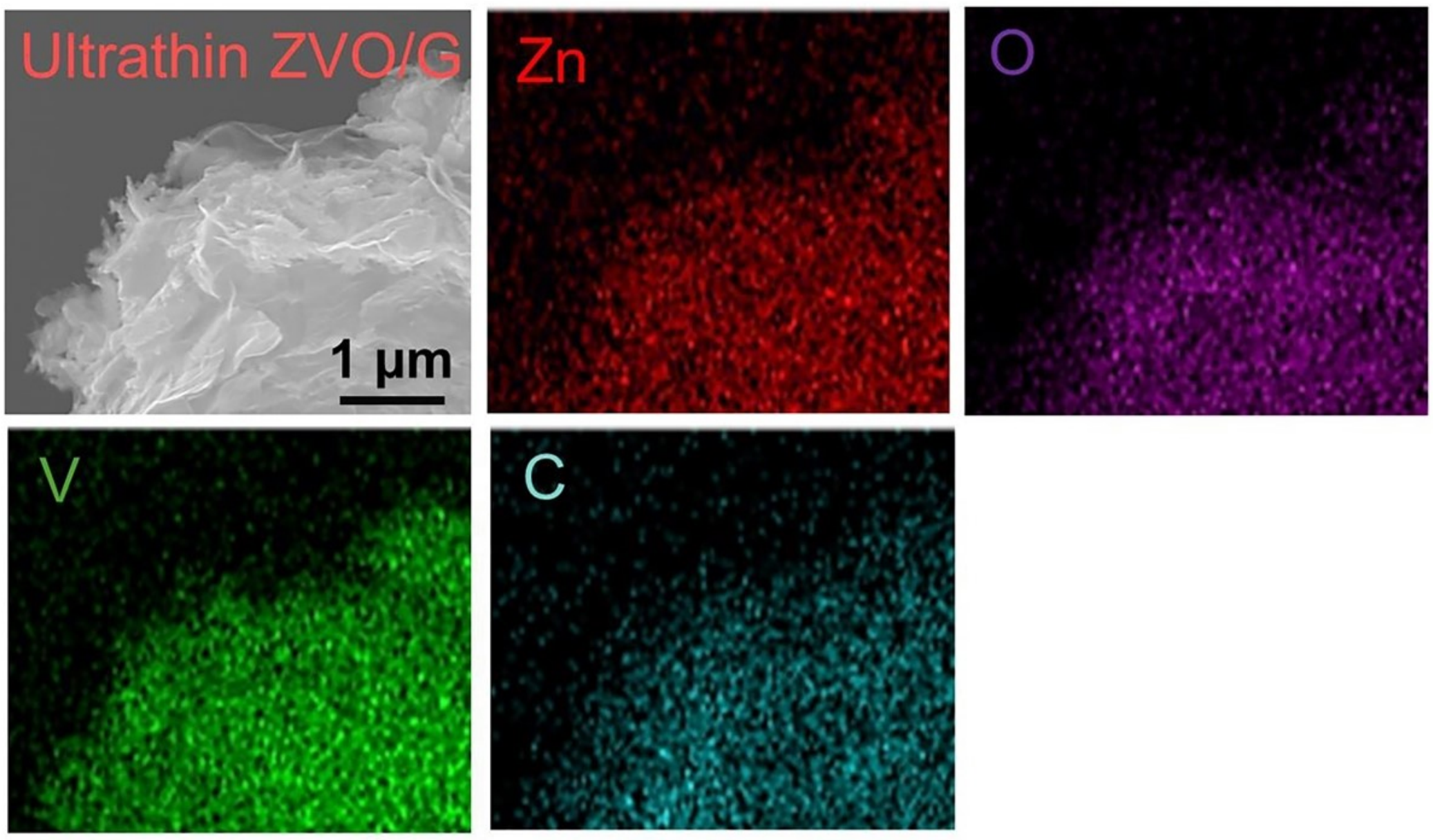

Fig. S15. Scanning transmission electron microscopy (STEM) and corresponding element mappings of ultrathin ZVO/G.

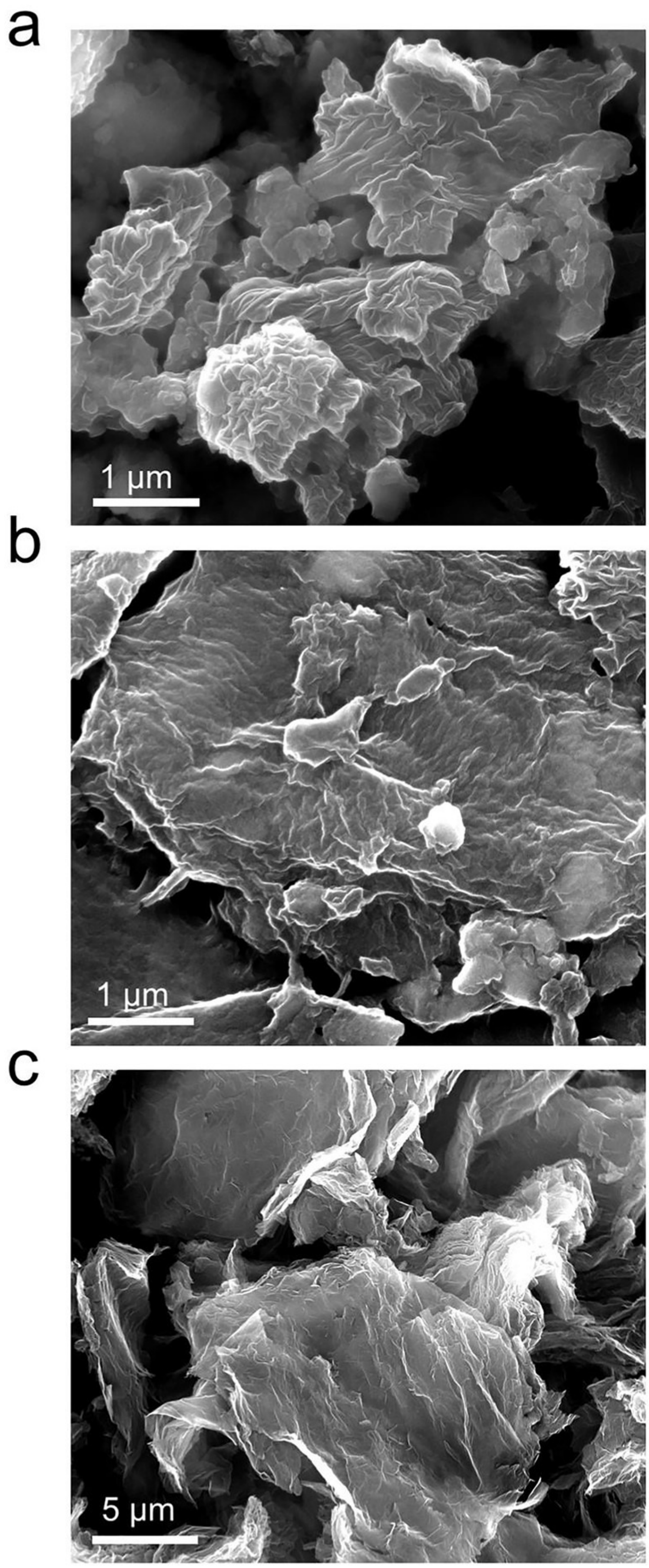

Fig. S16. SEM images of ultrathin ZVO/G nanosheets.

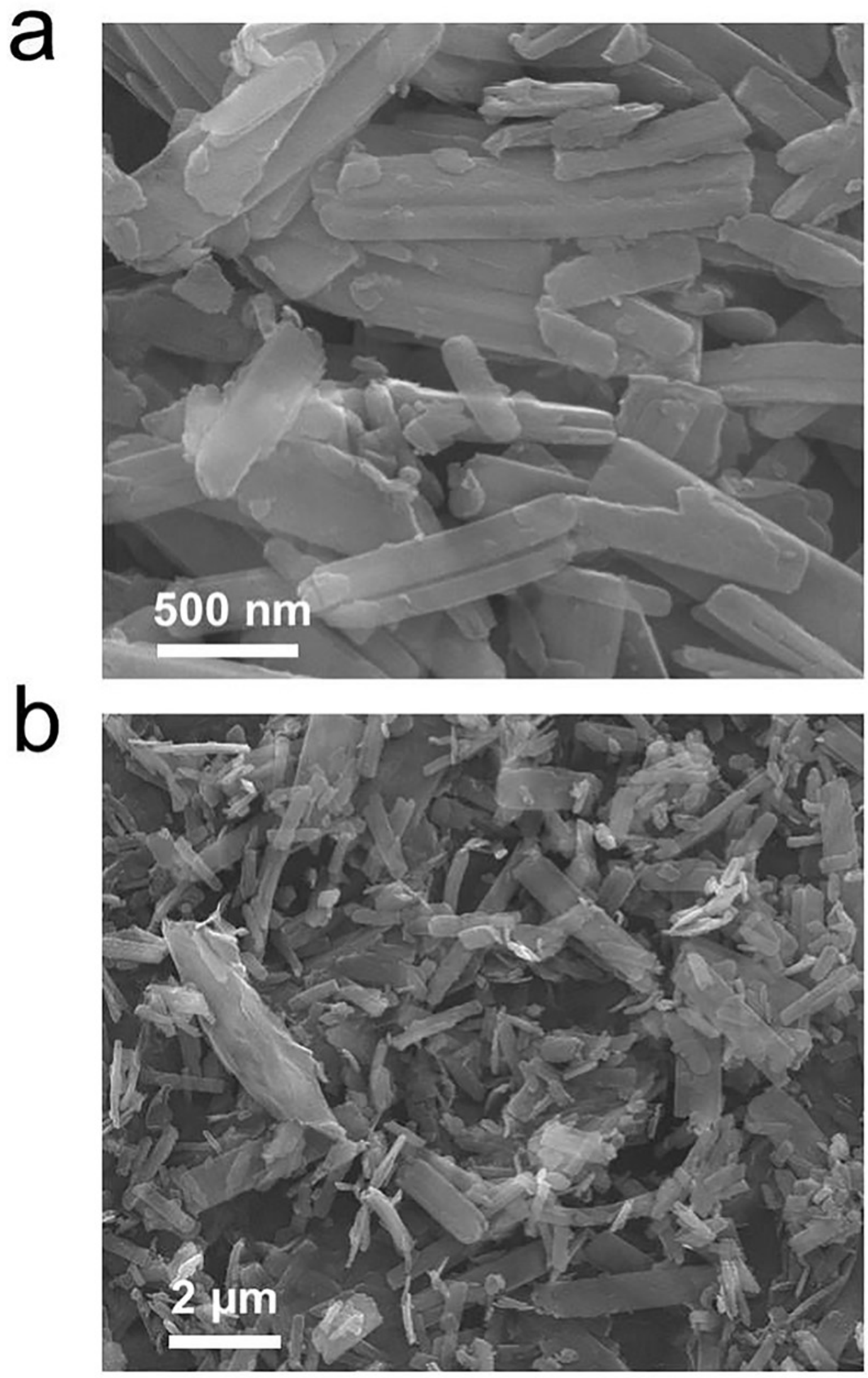

Fig. S17. SEM images of the mixture of ZVO and graphene (denoted as ZVO/G-BM) obtained by solid ball-milling.

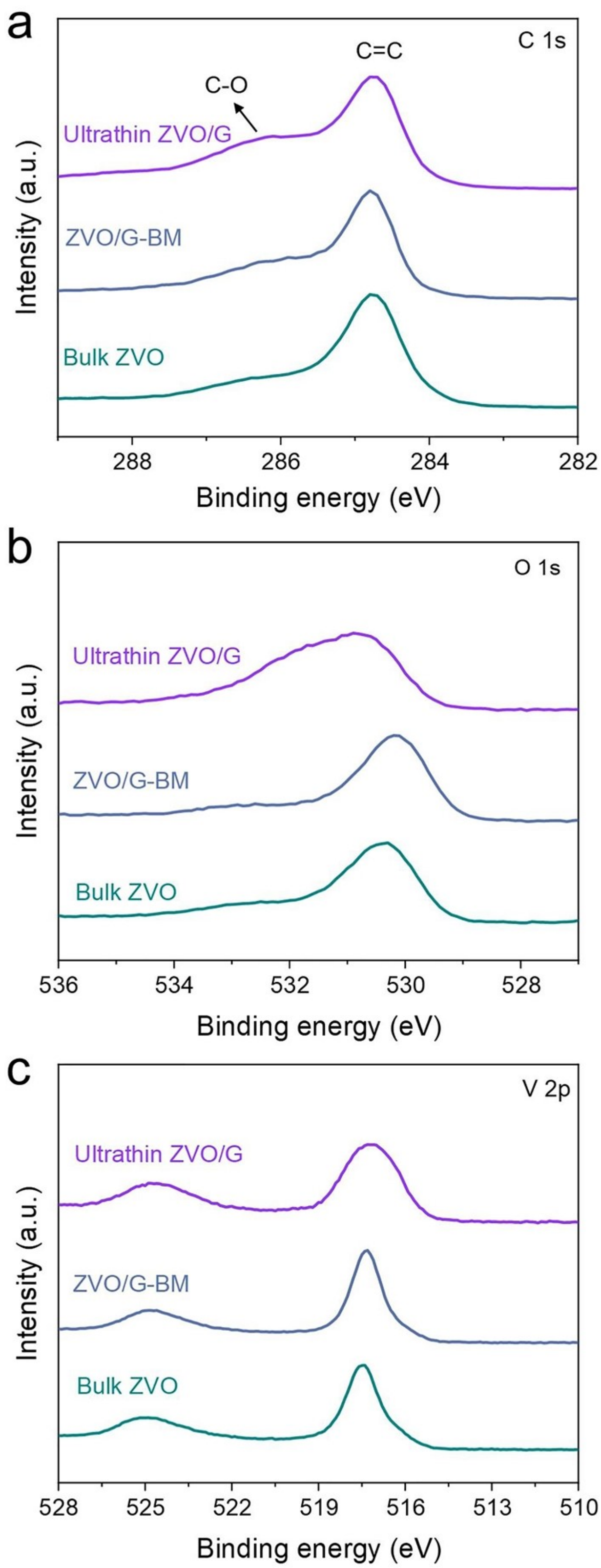

Fig. S18. XPS spectra of (a) C1s, (b) O1s and (c) V2p for ultrathin ZVO/G, ZVO/G-BM and bulk ZVO.

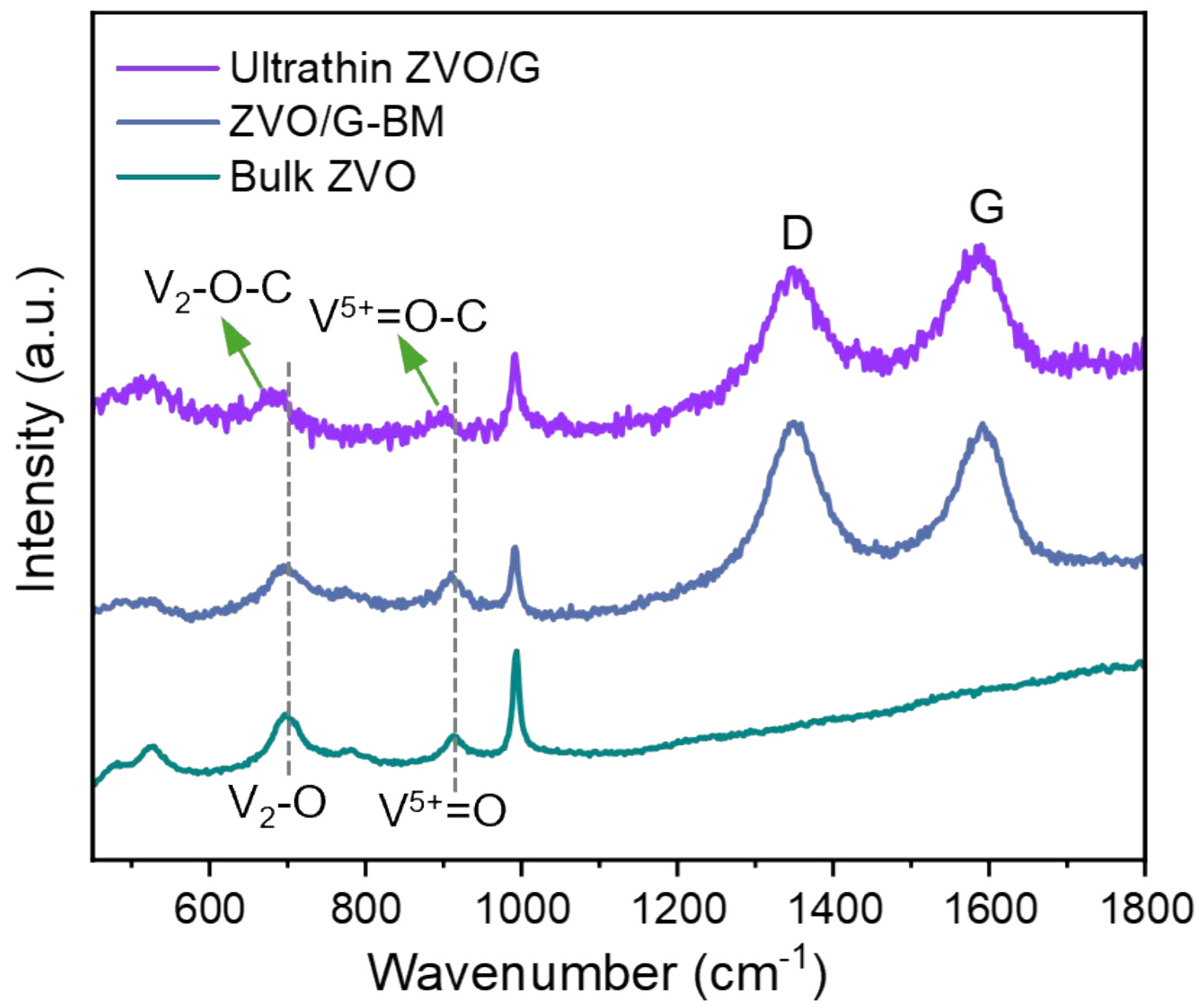

Fig. S19. Raman spectra of ultrathin ZVO/G, ZVO/G-BM and bulk ZVO.

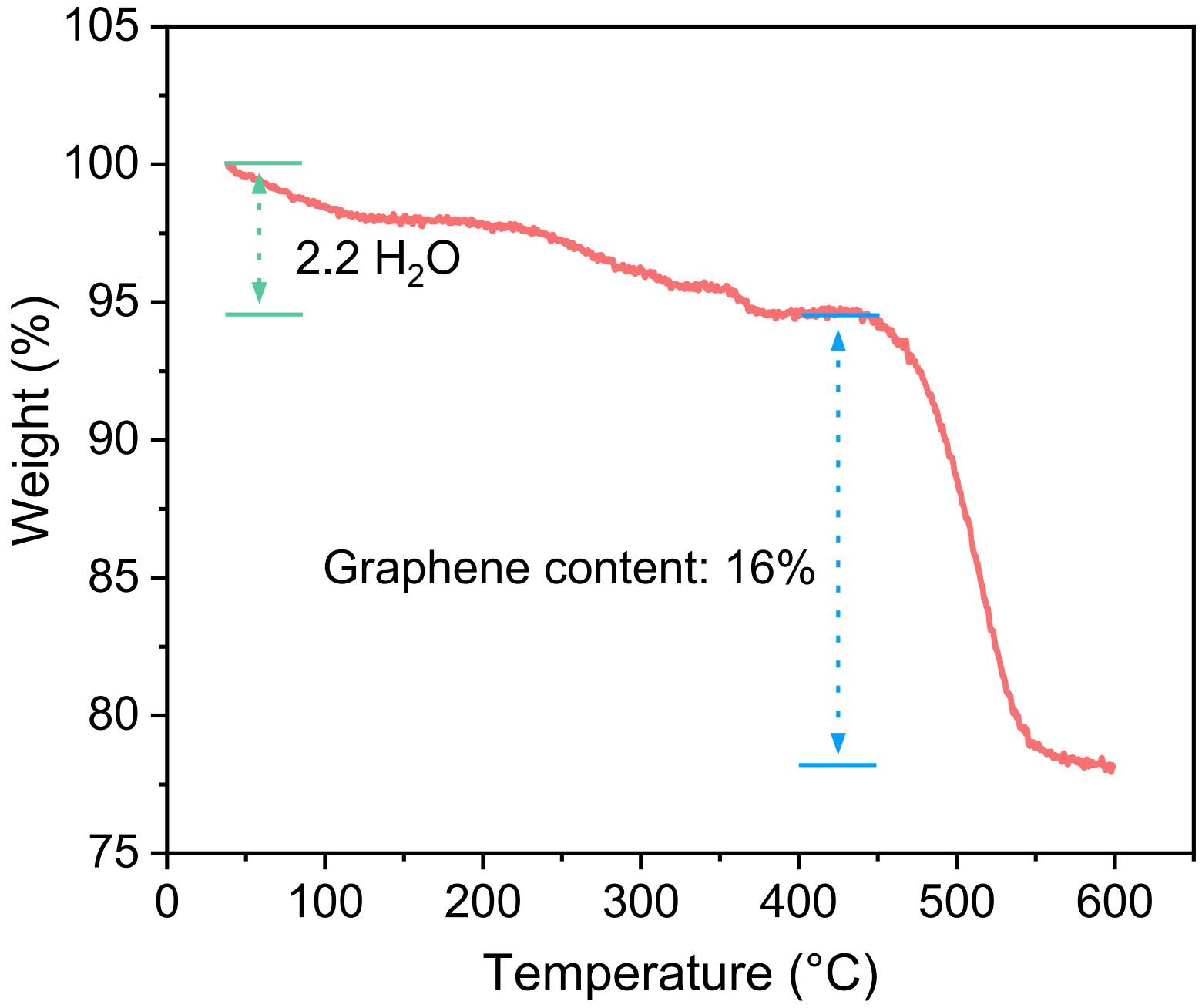

Fig. S20. Thermal gravimetric curve of ultrathin ZVO/G under air atmosphere at a heating rate of 5 °C min^{-1}.

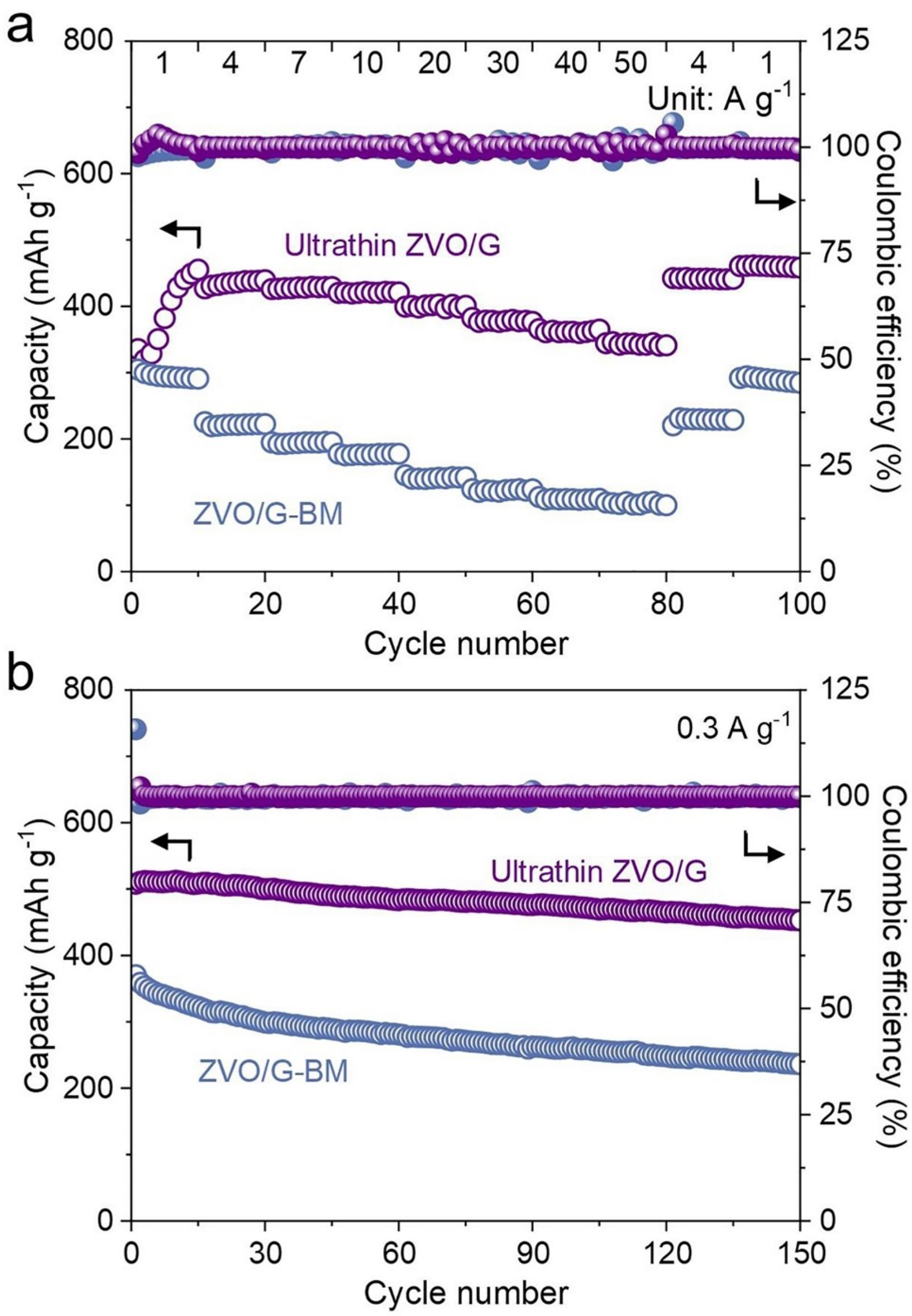

Fig. S21. (a) Rate performance and (b) cycling performances at low rate (0.3 A g^{-1}) for ultrathin ZVO/G and ZVO/G-BM.

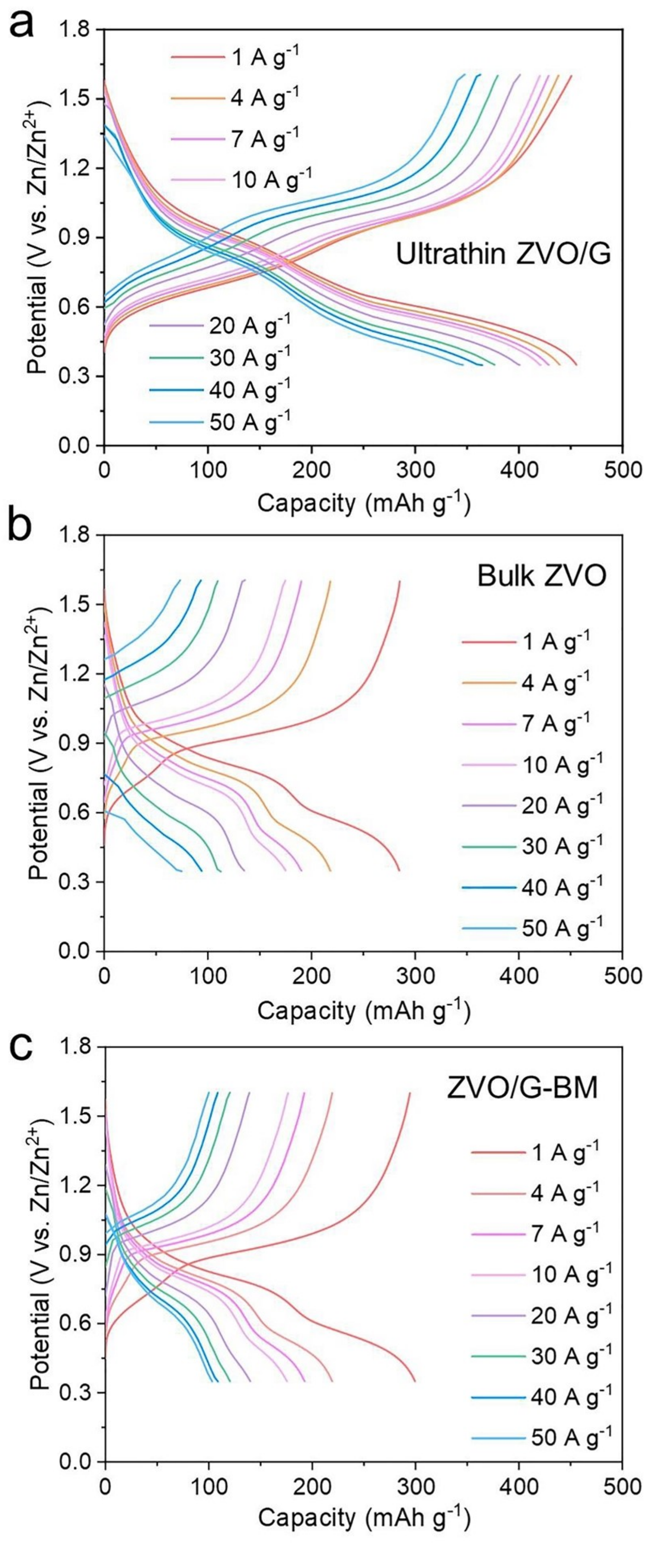

Fig. S22. Galvanostatic discharge/charge curves at different current densities in AZIBs with cathodes of (a) ultrathin ZVO/G, (b) bulk ZVO, and (c) ZVO/G-BM.

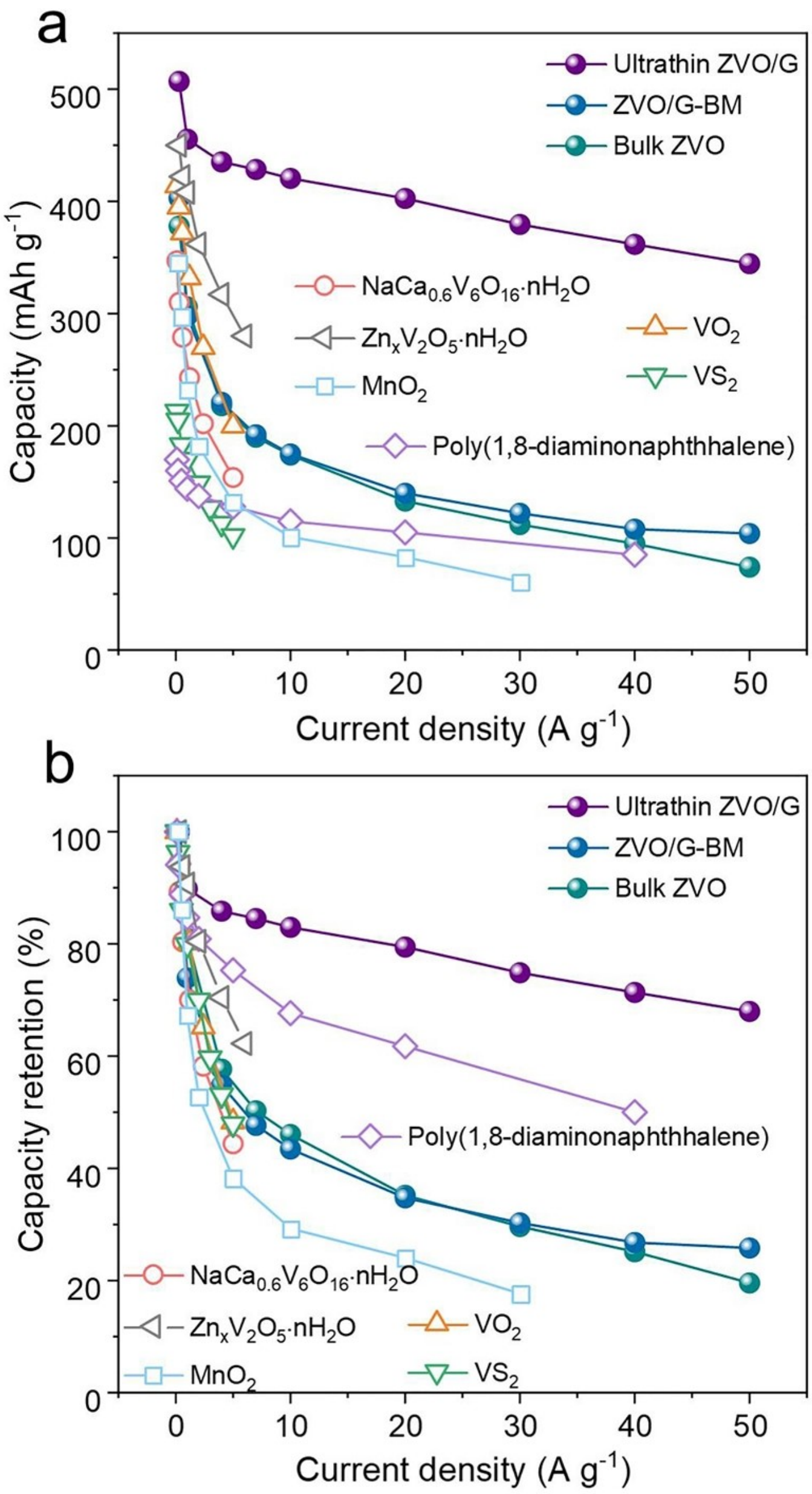

Fig. S23. Comparisons of (a) capacity or (b) capacity retention versus current density for ultrathin ZVO/G, ZVO/G-BM, bulk ZVO and other typical cathode materials in AZIBs[4-9].

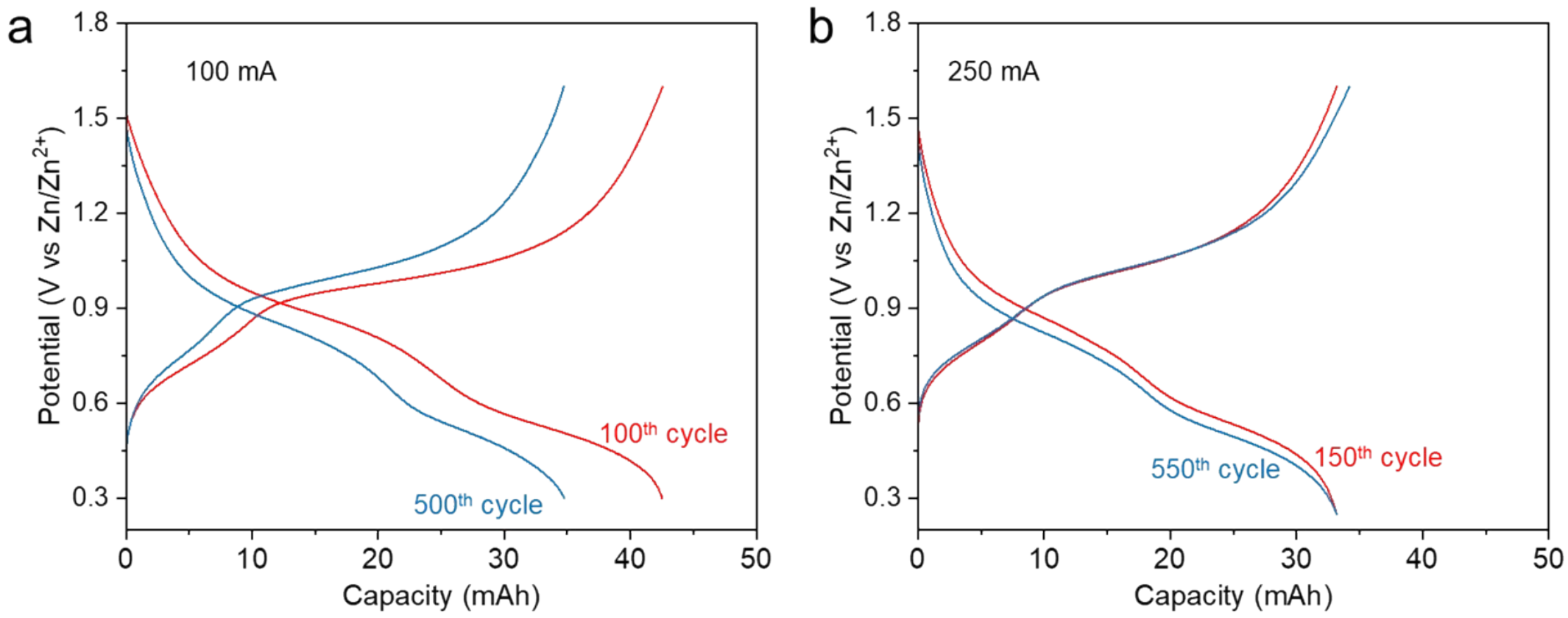

Fig. S24. Corresponding galvanostatic charge/discharge curves at certain cycles of a Zn||ultrathin ZVO/G pouch cell at currents of 100 mA and 250 mA.

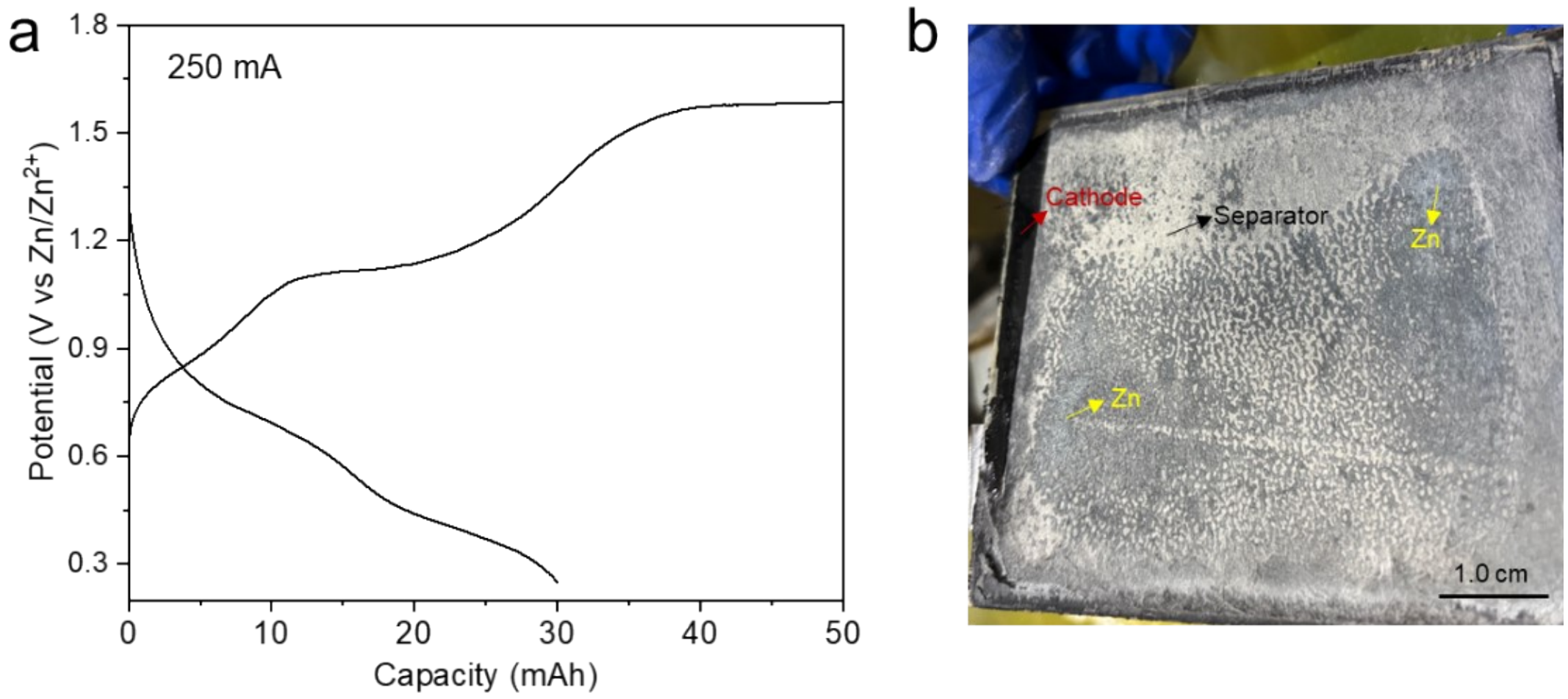

Fig. S25. (a) Galvanostatic charge/discharge curves at last cycle of the Zn||ultrathin ZVO/G pouch cell at current of 250 mA; (b) Optical images of separator in cycled Zn||ultrathin ZVO/G pouch cell.

Upon continued cycling of the pouch cell, the charge capacity was observed to be substantially higher than the discharge capacity (Fig. S25a), indicative of an internal short circuit that impedes proper charging. This failure mechanism was corroborated by disassembly of the cycled cell, which revealed the presence of Zn particles that had penetrated the separator (Fig. S25b).

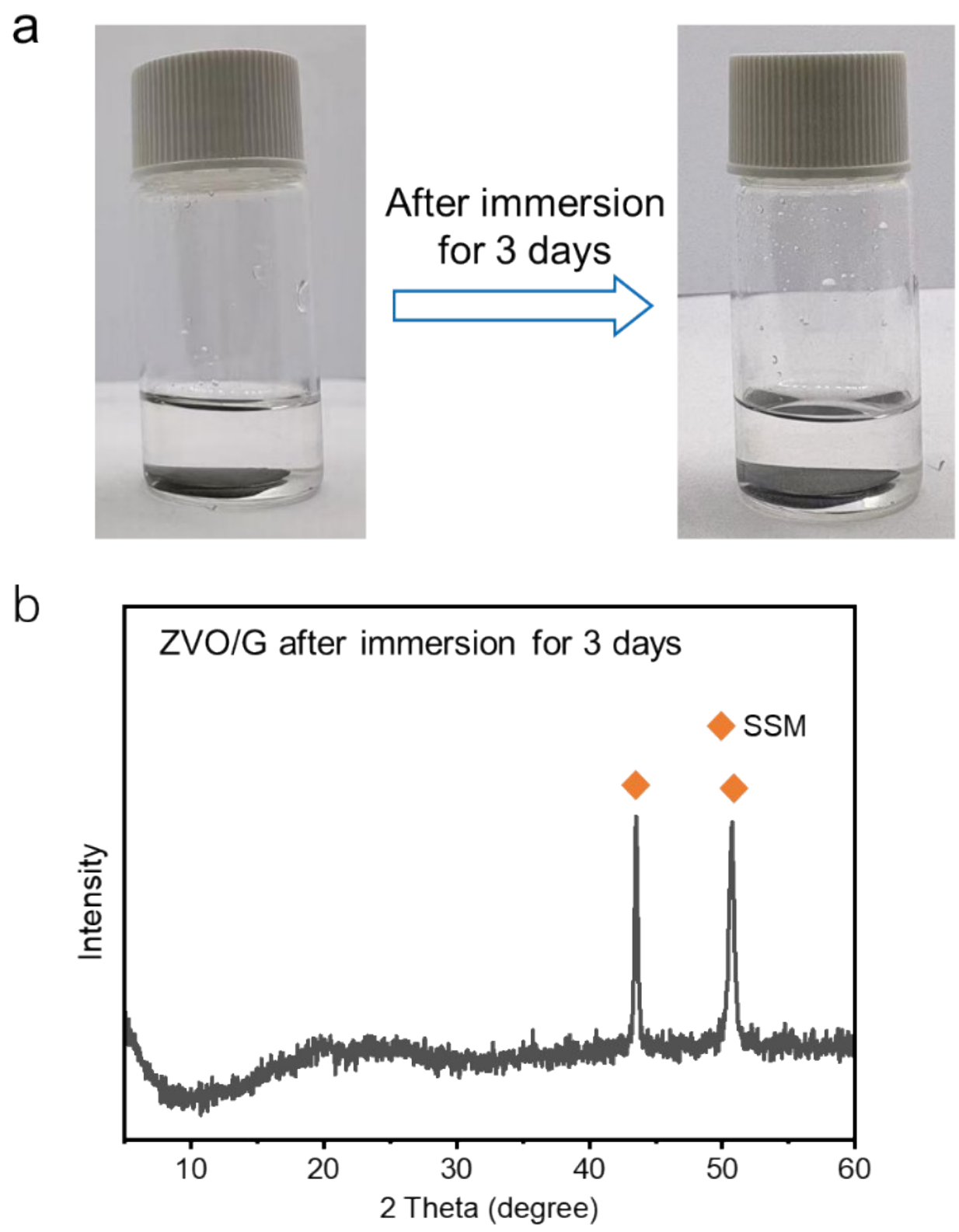

Fig. S26. (a) Optical images of ZVO/G cathode during immersion in 2M $Zn(OTf)_2$ for 3 days. (b) XRD pattern of ZVO/G cathode after immersion in 2M $Zn(OTf)_2$ for 3 days.

The ultrathin ZVO/G electrode was immersed in 2 M $Zn(OTf)_2$ electrolyte for 3 days to evaluate its structural stability. As shown in Fig. S26, the electrolyte remained colorless after immersion, and no detectable precipitates or secondary phases, such as $Zn_3(OH)_2V_2O_7{\cdot}2H_2O$, were observed on the electrode surface. These results demonstrate the excellent stability of the ultrathin ZVO/G electrode in the aqueous electrolyte.

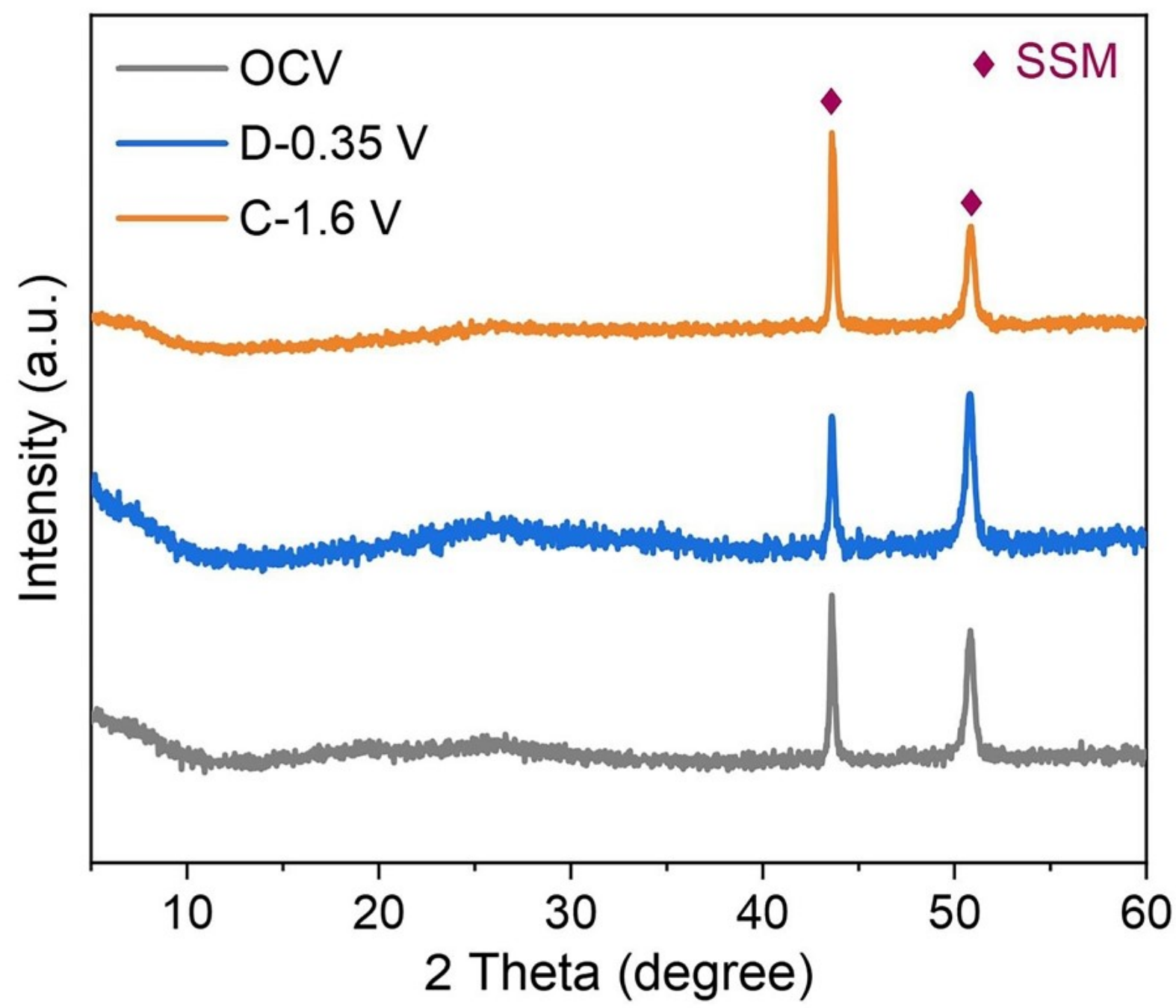

Fig. S27. XRD patterns of ultrathin ZVO/G cathodes at various states of charges.

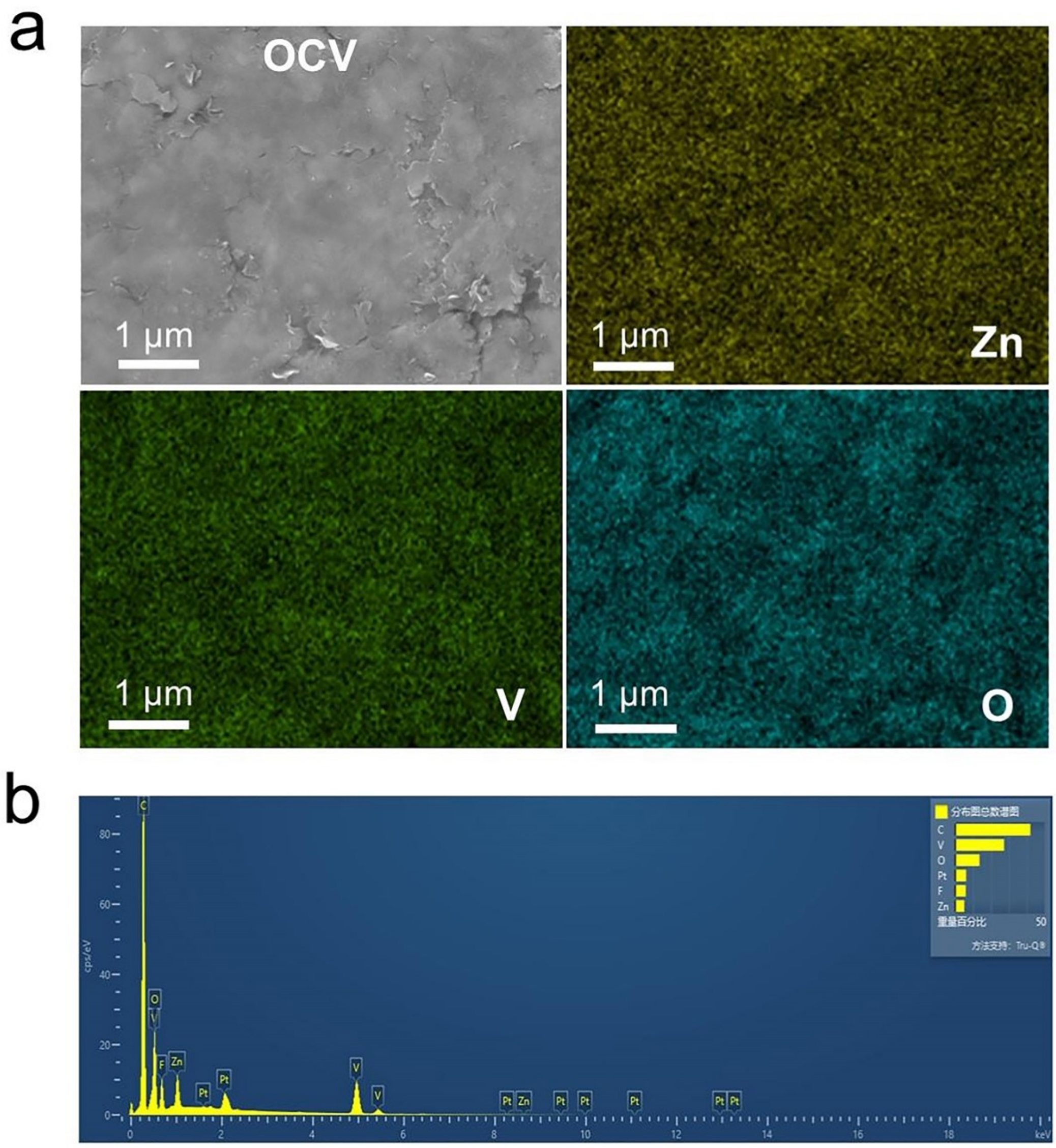

Fig. S28. (a) SEM image and corresponding element mappings, together with (b) energy-dispersive spectroscopy (EDS) analysis of ultrathin ZVO/G cathode at OCV.

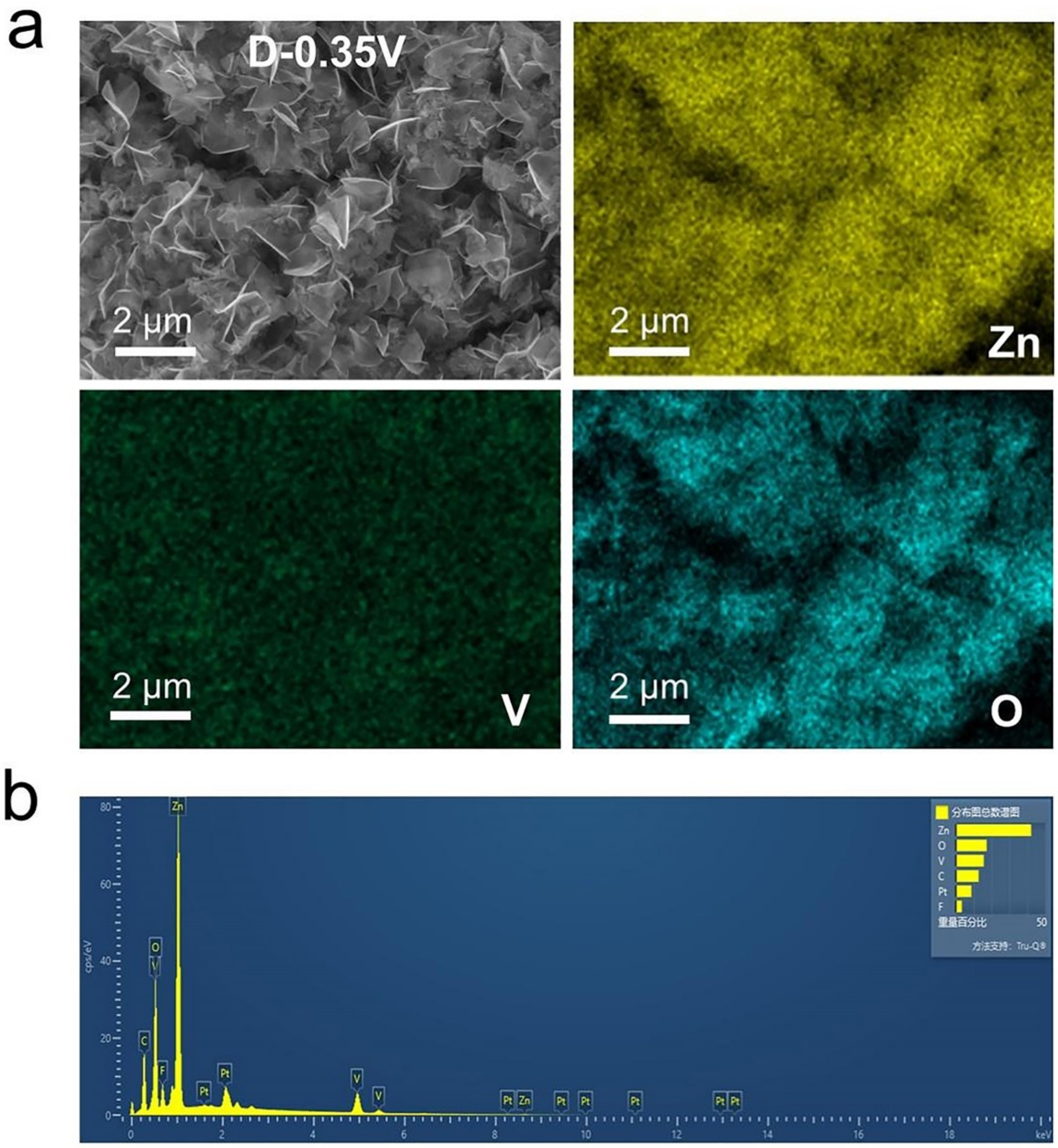

Fig. S29. (a) SEM image and corresponding element mappings, together with (b) energy-dispersive spectroscopy (EDS) analysis of ultrathin ZVO/G cathode at fully discharged state (D-0.35V).

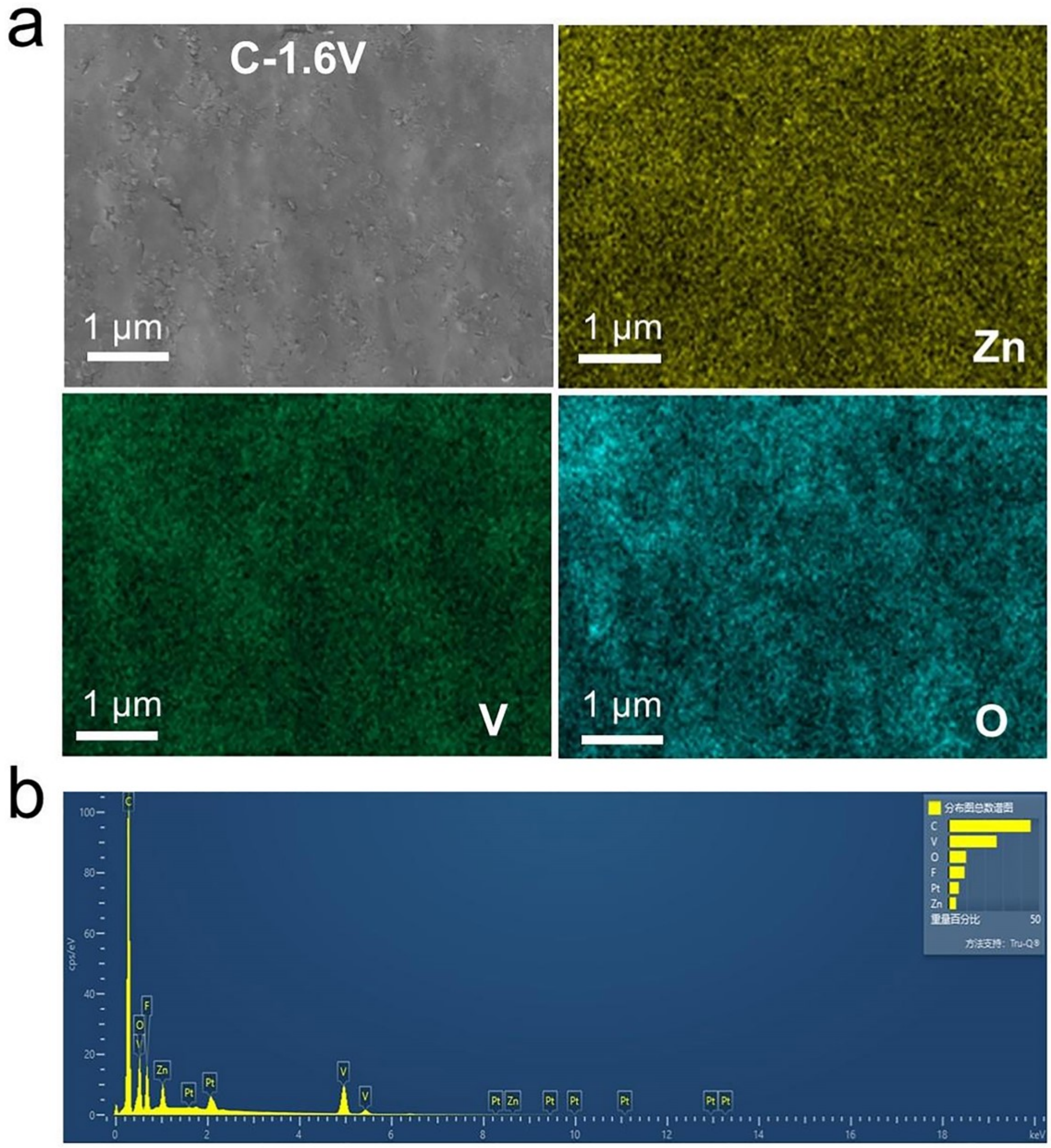

Fig. S30. (a) SEM image and corresponding element mappings, together with (b) energy-dispersive spectroscopy (EDS) analysis of ultrathin ZVO/G cathode at fully charged state (C-1.6 V).

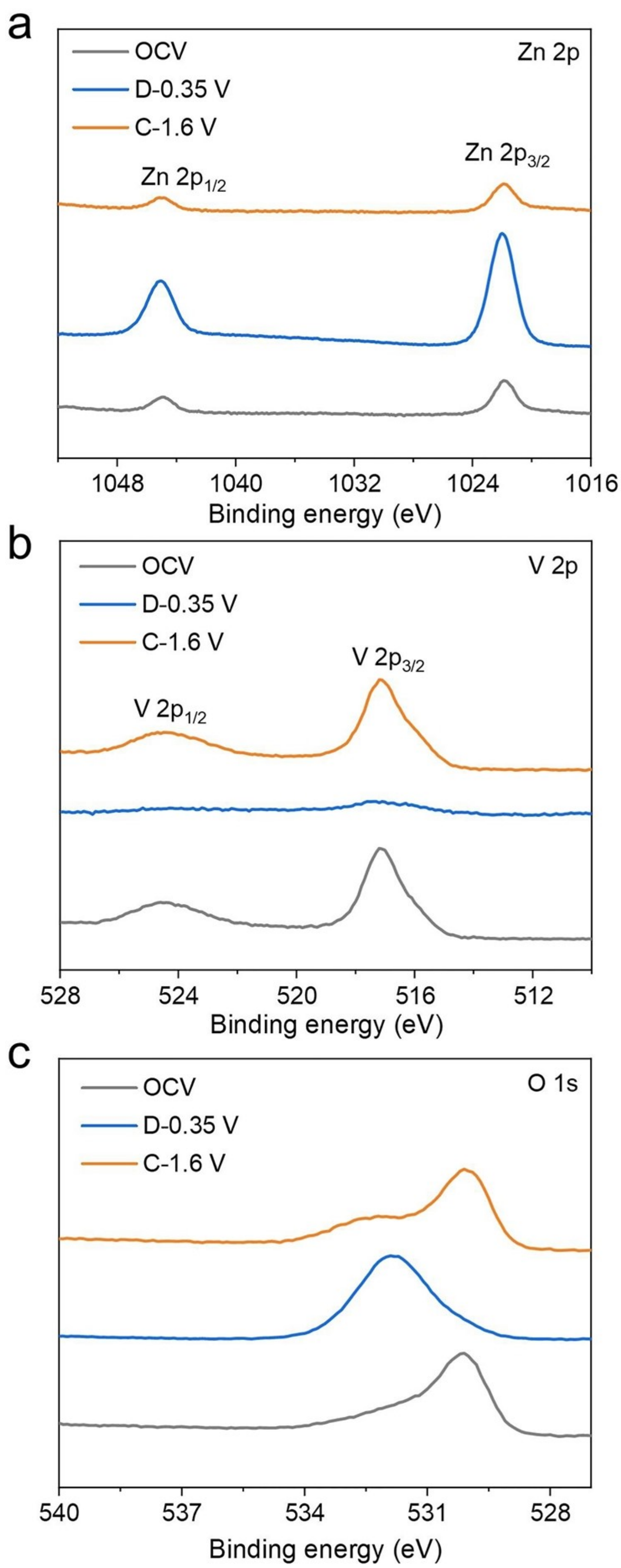

Fig. S31. XPS spectra of (a) Zn 2p, (b) V 2p, and (c) O 1s for ultrathin ZVO/G cathodes at various states of charge.

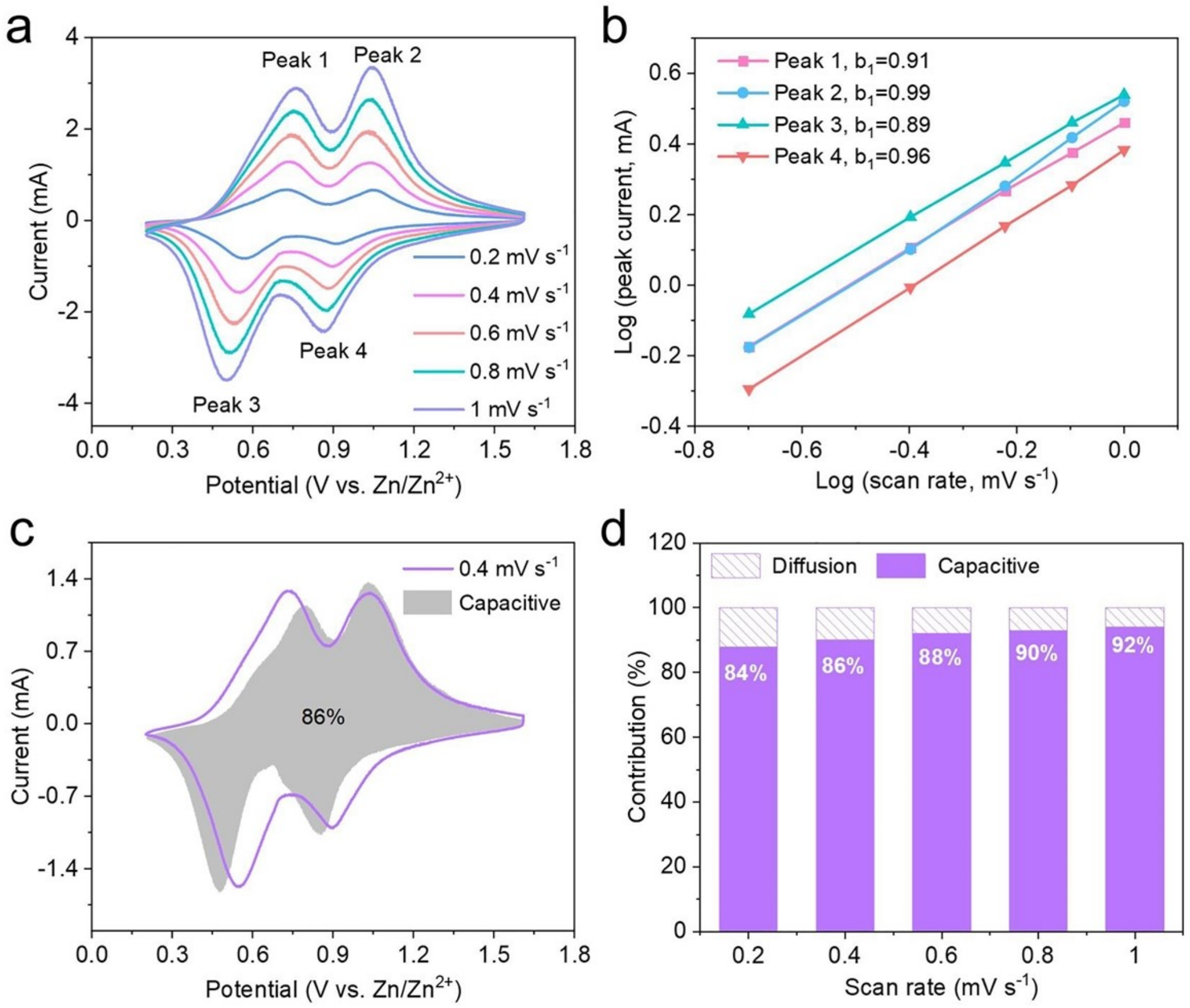

Fig. S32. Capacitive behavior analysis of ultrathin ZVO/G cathode. (a) CV curves at multiple scan rates (v) from 0.2 to 1 mV s^{-1}; (b) the corresponding plots of log (peak current) vs log (scan rate) at each cathodic and anodic peak; (c) capacitive contribution (grey portion) and diffusion contribution (white portion) at a scan rate of 0.4 mV s^{-1}; (d) the capacity contribution ratio at different scan rates.

Generally speaking, the peak current (i) of CVs can be related to scan rate by an empirical power-law relationship, which can be expressed as a combination of surface-controlled capacitive effects ($i_1=k_1v$) and diffusion-controlled Zn^{2+} (de)intercalation process ($i_2=k_2v^{1/2}$):[10]

$$i = k_1v+k_2v^{1/2} \approx av^b \quad (1)$$

where k_1, k_2, a and b are variable parameters; b=0.5 for a diffusion-controlled charge-transfer process and 1.0 for a surface-controlled capacitive process.

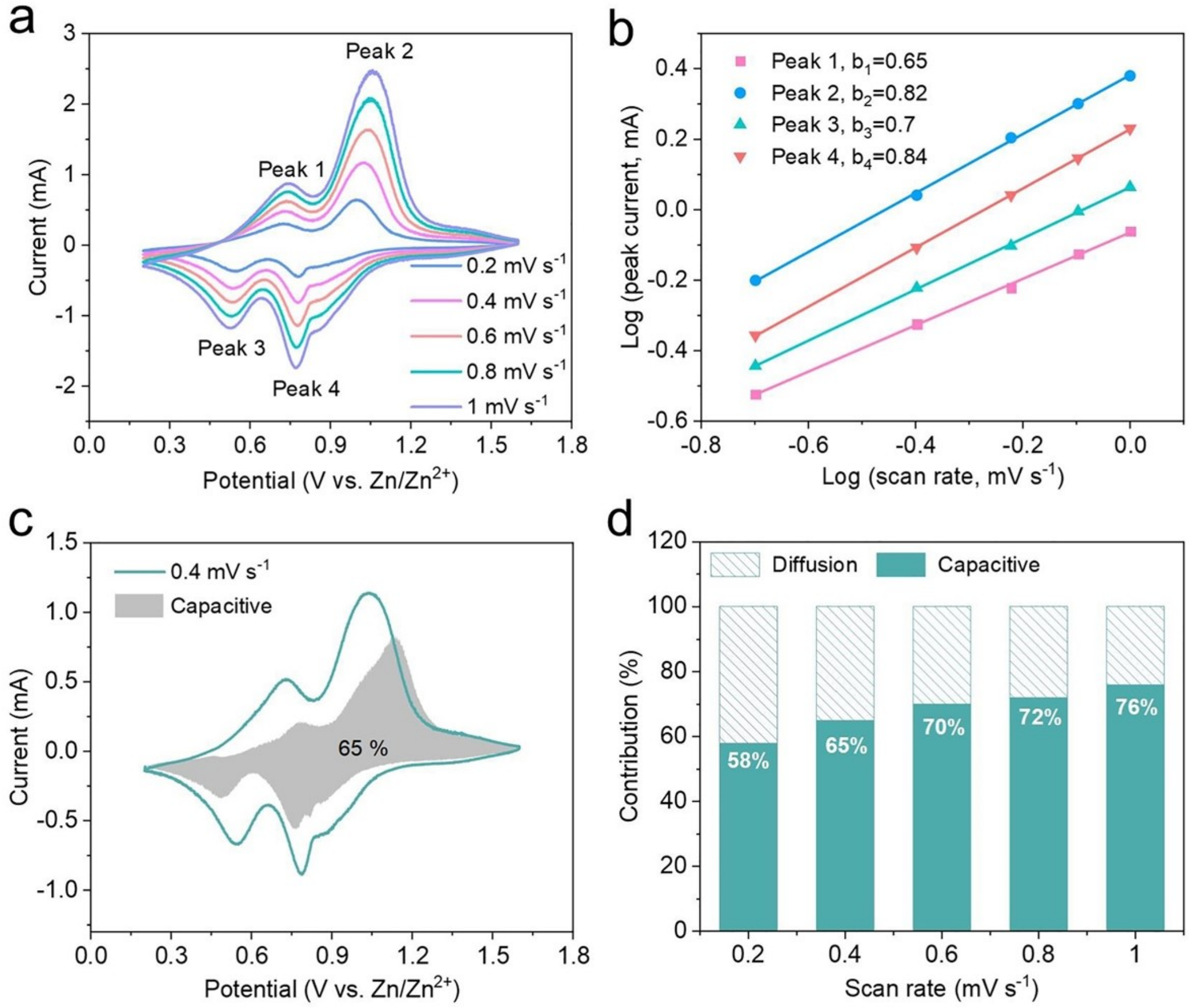

Fig. S33. Capacitive behavior analysis of bulk ZVO cathode. (a) CV curves at multiple scan rates (v) from 0.2 to 1 mV s^{-1}; (b) the corresponding plots of log (peak current) vs log (scan rate) at each cathodic and anodic peak; (c) capacitive contribution (grey portion) and diffusion contribution (white portion) at a scan rate of 0.4 mV s^{-1}; (d) the capacity contribution ratio at different scan rates.

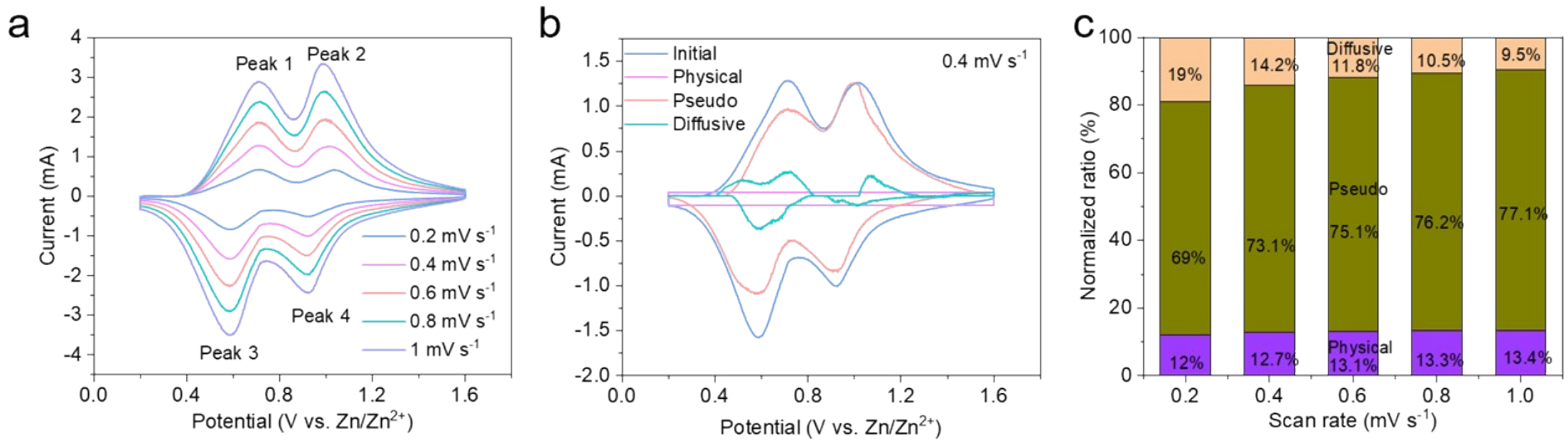

Fig. S34. (a) The CV curves of ultrathin ZVO/G cathode after de-residual. (b-c) details of three components contribution at various scan rates after de-residual.

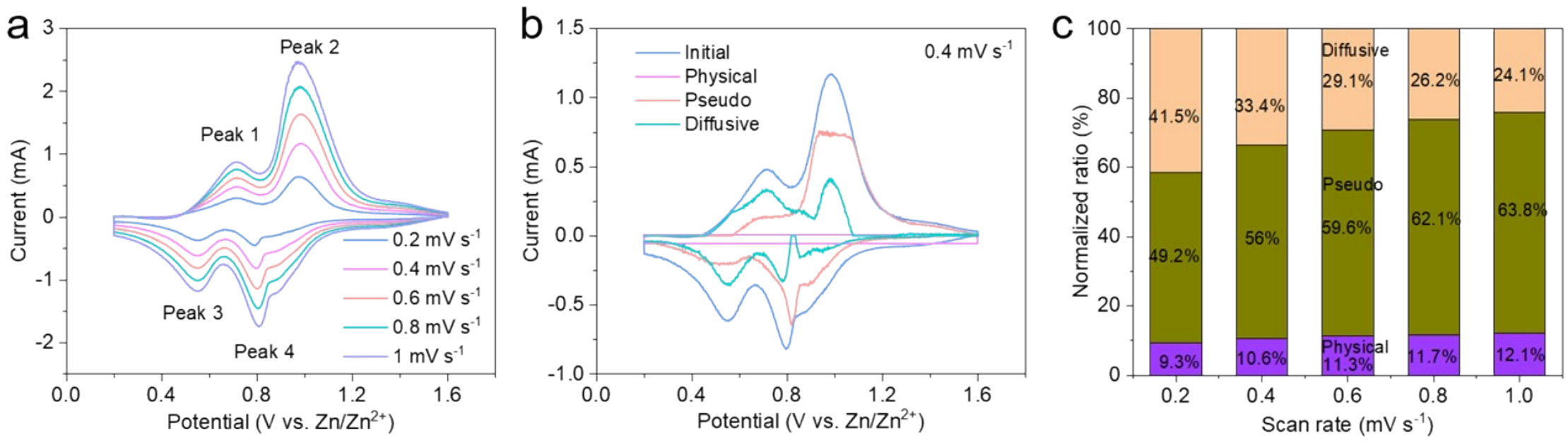

Fig. S35. (a) The CV curves of bulk ZVO after de-residual. (b-c) details of three components contribution at various scan rates after de-residual.

Table S1. Inductively Coupled Plasma Optical Emission Spectrometer results of ultrathin ZVO/G sample.

Number of test	Concentration of Zn (mg/L)	Concentration of V (mg/L)	Atomic ratio of Zn and V
1	5.995	31.53	0.9:6
2	6.492	34.13	0.9:6

Table S2. Test conditions for different cathode materials in the Ragone Plot.

Cathode materials	Electrolyte composition	Voltage window	Ref
MnO_2	1 M $ZnSO_4$ with 0.2 M $MnSO_4$	1.0-1.8V	32
MoS_2	PVA/$Zn(CF_3SO_3)_2$ (3M) hydrogel	0.2-1.3 V	33
$NaCa_{0.6}V_6O_{16}\cdot 3H_2O$	3 M $Zn(CF_3SO_3)_2$	0.4-1.5V	34
$Zn_{0.1}V_2O_5\cdot nH_2O$	2 M $ZnSO_4$	0.25−1.70 V	35
$VO_2\cdot 0.45H_2O$	3 M $Zn(CF_3SO_3)_2$	0.35−1.35 V	36
Poly(1,8-diaminonaphthalene)	0.5 M $Zn(CF_3SO_3)_2$	0-1.6V	37
Zinc hexacyanoferrates	1 M $ZnSO_4$	0.8-2V	38
VS_2	PVA/$Zn(CF_3SO_3)_2$ (2.5M) hydrogel	0.4-1.0V	39
Ultrathin ZVO	2 M $Zn(CF_3SO_3)_2$	0.35-1.6 V	This work

References

1. K. Zhu, W. Jiang, Z. Wang, W. Li, W. Xie, H. Yang and W. Yang, *Angew. Chem. Int. Ed.*, 2023, **62**, e202213368.
2. D. Zhang, X. Liu, X. Zhang, C. Zhang, C. Cai, H. Bi, Y. Du, X. Qin, A. Peng and J. Huang, *NPJ Comput. Mater.*, 2024, **10**, 293.

3. A. Hjorth Larsen, J. Jørgen Mortensen, J. Blomqvist, I. E. Castelli, R. Christensen, M. Dułak, J. Friis, M. N. Groves, B. Hammer and C. Hargus, *J. Phys.: Condens. Matter*, 2017, **29**, 273002.
4. T. Xiong, Z. G. Yu, H. Wu, Y. Du, Q. Xie, J. Chen, Y. W. Zhang, S. J. Pennycook, W. S. V. Lee and J. Xue, *Adv. Energy Mater.*, 2019, **9**, 1803815.
5. K. Zhu, T. Wu and K. Huang, *Adv. Energy Mater*., 2019, **9**, 1901968.
6. K. Zhu, T. Wu, W. van den Bergh, M. Stefik and K. Huang, *ACS nano*, 2021, **15**, 10678-10688.
7. K. Zhu, T. Wu, S. Sun, W. van den Bergh, M. Stefik and K. Huang, *Energy Storage Mate.* , 2020, **29**, 60-70.
8. L. Yan, Q. Zhu, Y. Qi, J. Xu, Y. Peng, J. Shu, J. Ma and Y. Wang, *Angew. Chem. Int. Ed.*, 2022, **61**, e202211107.
9. Y. Tan, S. Li, X. Zhao, Y. Wang, Q. Shen, X. Qu, Y. Liu and L. Jiao, *Adv. Energy Mater.*, 2022, **12**, 2104001.
10. V. Augustyn, P. Simon and B. Dunn, *Energy Environ. Sci.*, 2014, **7**, 1597-1614.
